# Spontaneous evolution of human skin fibroblasts into wound-healing keratinocyte-like cells

**DOI:** 10.7150/thno.31526

**Published:** 2019-07-09

**Authors:** Fang Zhang, Dandan Zhang, Kai Cheng, Zaixin Zhou, Shupeng Liu, Liang Chen, Yijun Hu, Chuanbin Mao, Shanrong Liu

**Affiliations:** 1 Department of Laboratory and Diagnosis, Changhai Hospital, Second Military Medical University, Shanghai 200433, China.; 2 No. 73901 troops, Shanghai 200439, China.; 3 Clinical Research Center, Changhai Hospital, Second Military Medical University, Shanghai 200433, China.; 4 Department of Chemistry and Biochemistry, Stephenson Life Sciences Research Center, Institute for Biomedical Engineering, Science and Technology, University of Oklahoma, 101 Stephenson Parkway, Norman, Oklahoma 73019-5300, USA.

**Keywords:** cellular conversion, time-dependent, fibroblasts, keratinocyte-like cells, wound healing

## Abstract

Producing keratinocyte cells (KCs) in large scale is difficult due to their slow proliferation, disabling their use as seed cells for skin regeneration and wound healing. Cell reprogramming is a promising inducer-based approach to KC production but only reaches very low cellular conversion. Here we reported a unique cellular conversion phenomenon, where human skin fibroblasts (FBs) were spontaneously converted into keratinocyte-like cells (KLCs) over the time without using any inducers.

**Methods**: FBs were routinely cultured for more than 120 days in regular culture medium. Characteristics of KLCs were checked at the molecular and cellular level. Then the functionality and safety of the KLCs were verified by wound healing and tumorigenicity assay, respectively. To identify the mechanism of the cell conversion phenomenon, high-throughput RNA sequencing was also performed.

**Results**: The global conversion started on day 90 and reached 90% on day 110. The KLCs were as functional and effective as KCs in wound healing without causing oncogenicity. The conversion was regulated via a PI3K-AKT signaling pathway mediated by a long non-coding RNA, LINC00672. Modulating the pathway could shorten the conversion time to 14 days.

**Conclusion**: The discovered FBs-KLCs conversion in the study might open a new avenue to the scalable production of cell sources needed for regenerating skins and healing large-area wounds.

## Introduction

Burns are a major global public health problem due to their significant morbidity and mortality 1 and high treatment cost 2, 3. Skin defect caused by burns is a great challenge in reconstructive surgery. Current standard for permanent closure of full-thickness burn wounds is autologous split-thickness skin grafting 4. However, in the extensive burns, the available donor sites are limited and can hardly meet the large-scale needs of skin grafts, limiting the patient's survival chance 5. In order to provide a highly increased grafting expansion ratio and reduce the burdens of donor sites, many advanced technologies, such as MEEK grafting 6, ReCell 7 and Epicel 8 techniques, were developed. Although these technologies significantly reduced the healing time of both the donor sites and the burn wounds, they were limited by the shortage of skin sources. A promising alternative strategy is to implant keratinocyte cells (KCs) as a cell source for wound healing 9, 10. However, obtaining a large amount of KCs is still a challenge because KCs proliferate slowly. Cell reprogramming has been proposed to obtain KCs in a large quantity from induced pluripotent stem cells (iPSCs). However, it required the delivery of transcription factors as inducers (Klf4, Sox2, Oct4, and c-Myc) into FBs and the resultant iPSCs could be differentiated into KCs. Such approach has a very low cellular conversion rate 11, 12. Therefore, it is still difficult to produce functional KCs in a large scale.

To seek a new facile approach to the production of KCs, we made an attempt to convert FBs, which can be produced in a large scale due to their natural fast proliferation, into KCs. Surprisingly, we discovered a unique cellular conversion phenomenon, where human skin fibroblasts (FBs) were spontaneously converted into keratinocyte-like cells (KLCs) in the regular DMEM in a time-dependent manner, bypassing the need of forced expression of the inducers (transcription factors and miRNA 13). The converted KLCs were as functional and effective as KCs in wound healing, and did not cause oncogenicity. To understand the underlying mechanisms for the FBs-KLCs conversion, high-throughput mRNA and lncRNA sequencing was performed. By the ceRNA analysis, we identified that this time-dependent epidermal conversion was regulated via the PI3K-AKT signaling pathway mediated by the LINC00672.

## Methods

### Reagents and antibodies

Rabbit monoclonal antibodies against Oct4 and K6, rabbit polyclonal antibody against TLR4 and mouse monoclonal antibody against α-SMA were purchased from Abcam Biotechnology (Dallas, TX, USA). Rabbit monoclonal antibodies against vimentin, AKT3, PTEN and K17, and mouse monoclonal antibodies against K19 and β-actin were obtained from Cell Signaling Technology (Beverly, MA, USA). Donkey anti-rabbit-IgG and anti-mouse-IgG antibodies (horseradish peroxidase-labeled) were obtained from Santa Cruz Biotechnology (Dallas, TX, USA). Vimentin antibody used for flow cytometry was purchased from eBioscience Systems (San Diego, CA, USA). Inhibitors of AKT3, TLR4 and PTEN were purchased from MedChem Express (Monmouth, NJ, USA). Trizol reagent was purchased from Invitrogen (Carlsbad, CA, USA). siRNAs of LINC00672 were bought from Sangon Biotech Co., Ltd (Shanghai, China).

### Cell isolation and culture

Human primary FBs and KCs were isolated from the foreskin of three 10-20 year-old males. The skin was washed with chlorhexidine and PBS for three times and then cut into 0.5×0.5 cm split-thickness sections. The skin was immersed in Dispase II (Gibco) for 16 h at 4°C, and epidermal layer was separated from the dermal layer with tweezers. The epidermis and dermis were digested with Trypsase (Invitrogen) and collagenase I (Gibco) for 30 min at 37°C separately, and grinded into a single-cell suspension through a mesh funnel. Culturing of FBs was carried out in DMEM (Corning) containing 10% FBS (Gibco). We passaged the cells about every seven days when the cell density reached about 90%. After every passaging, we saved 40% cells and continued to culture them. Sodium acetate (3M) was added into the culture media after 90 days. KCs were cultured in Coating matrix (Gibco) coated dished with serum free Defined K-SFM (Gibco). Hep3B cells were received as a gift from the Eastern Hepato-Biliary Hospital, Second Military Medical University, Shanghai. Hep3B, FBs, and KLCs were enriched in spheroids cultured using DME/F12 supplemented with FGF, IGF and EGF as described previously 14.

All cells were cultured in gelatin-coated 10-cm cell culture dishes, which were placed in the incubators (5% CO_2_, 37°C) with media changes every third day (Hep3B/KCs) or every fourth day (FBs/KLCs). All the cells were cultured without antibiotics. Inhibitors of AKT3, TLR4, PTEN and siRNAs of LINC00672 were used according to the supplier's protocols.

### Animals

Nude mice (Six-week-old and male) with a body weight of 18-20g were obtained from the Experimental Animal Centre of SMMU. The animals were used under the protocol defined by the guidelines of the Institutional Animal Care and Use Committee (IACUC) of the SMMU.

### Experimental wound preparation and analysis

Four groups of randomly picked nude mice were used to evaluate wound healing with n=5 for each group. Full-thickness skin defect measuring 1.0×1.5 cm was created on the dorsal skin, as described by Wu et al 15. The treatment protocol was as follows: (1) Control group: 0.5 ml saline was transplanted onto the whole wound topically; (2) KLCs group: 0.5 ml KLCs (1×10^8^/ml) were transplanted onto the whole wound topically; (3) KCs group: 0.5 ml KCs (1×10^8^/ml) were transplanted onto the whole wound topically; and (4) FBs group: the wound was transplanted with 0.5 ml FBs (1×10^8^/ml ) on the whole wound. The treatment was given every day. After treatment with cells or saline, the wound was covered with vaseline gauze and sterile gauze as clinical practice. The mice were intraperitoneally anesthetized with 0.1ml 1% sodium pentobarbital before the change of the fresh wound dressing and taking photographs. The wound healing percentage was calculated on day 6, 12, 18, and 28 by the following equation 2. The percentage of wound healing =(A1-A2)/A1×100%, where A1 and A2 are the area of original and actual (at a given time point) wound, respectively.

### Tumorigenicity assay

Nude mice were grouped into two (KLCs versus KCs) with each having randomly picked 5 mice. KLCs and KCs were suspended (5×10^6^ cells per 100 µl) in DMEM without FBS. We injected 100 µl of the cell suspension (5×10^6^ cells) into the subcutaneous tissue of mice. 45 days after cell transplantation, mice were sacrificed and neoplasms were dissected for histopathological detection 16.

### Haematoxylin and eosin (H&E) staining

For histopathological analysis, formaldehyde-fixed, paraffin-embedded skin sections were affixed and H&E staining was performed as previously described 17. Histopathological changes were observed using a light photomicroscope (Leica).

### Immunofluorescence analysis

4% paraformaldehyde was used to fix the cells for 10 min, which were further permeabilized with 0.4% Triton X-100. Primary antibodies K6, vimentin, α-SMA and Oct4 were added into the cells for incubating overnight at 4°C. Then the cells were allowed to interact with donkey anti-mouse or anti-rabbit IgG antibodies for 1 h, and then the cell nuclei were counterstained with DAPI solution for an additional 20 min at 37°C 18. Finally, the images were captured with a fluorescence microscope (Leica).

### Immunohistochemistry for K6

Immunohistochemistry was performed using the human special antibody K6. Briefly, tissue sections were deparaffinized and incubated with K6 overnight at 4°C. They were then washed by PBS, followed by staining with donkey anti-mouse or anti-rabbit IgG antibodies. Diaminobenzidine was used to visualize the labeling and hematoxylin was used to counterstain the slides.

### Flow cytometry

To quantify cells with positive vimentin expression in cytoplasm, flow cytometry was performed. Briefly, FBs, KLCs and KCs were harvested and fixed with a fixation buffer (eBioscience) at 4°C for 30 min. Then cells were penetrated with a permeabilization buffer (eBioscience) and further incubated with the corresponding antibodies at 4°C over the night. After incubation, the cells were washed and formed a suspension of 200 μl. Flow cytometry was used to analyze the cell suspension by a FACScan (Miltenyi Biotech, Germany).

### Image flow cytometry analysis

The cell digestion, fixation, transmembrane and incubation procedures were the same as flow cytometry assays according to the previous report 19. Cells were analyzed by a FlowSight cytometer (Merck Millipore, Germany).

### qRT-PCR analysis

RNA extraction and qRT-PCR assays were carried out following a reported method 20. Briefly, after the collection and washing of the cells, total RNA was purified using Trizol reagent and reverse transcribed in a 20μL oligo (dT) system. The resultant cDNA amplified by PCR under the conditions listed below: at 95°C for 30 seconds for pre-degenerated, then at 95°C for 5 seconds, and at 60°C for 30 seconds for 45 cycles using LightCycler 480 II (Roche USA). The primer sequences were presented in Table S3.

### Western blot assays

The proteins were extracted from about 1×10^6^ cells by using 200 μL cell lysis buffer. The resultant suspension was subjected to centrifugation (12,000 rpm) at 4°C for 15 min. BCA assay was used to quantify the protein concentrations in the supernatants. Western blot analysis was performed to evaluate vimentin, α-SMA, K6, K17, K19 and β-actin as in our previous study 21.

### Cell migration assays

Invasion assays: FBs, KLCs and KCs were seeded onto the filters of Matrigel-coated transwell plates. Cells invasion was assessed at 48 h after seeding 22. EGF was added to the lower compartment for KCs.

Scratch wound assays: FBs, KLCs and KCs were incubated in a 12-well plate at a seeding density of 2×10^5^ cells per well. Wound scratches were created with 200 μl plastic filter tips. The culture plates were washed three times to eliminate the dislodged cells. After 48 h, images were captured using a light microscope (Leica) and the migrated cells were calculated.

### CCK8 assays

FBs, KLCs and KCs were seeded in a 96-well plate and allowed to grow with adherence. Cell Counting Kit-8 (CCK8) reagent was added into each well, and the absorbance at 12, 24, and 48 h was measured at 450 nm using Varioskan Flash (Thermo fisher, USA).

### Whole Transcriptome Sequencing and Data Analysis

RNA extracted by Trizol reagent (Invitrogen) with RIN>6.0 was utilized to construct a rRNA depletion library (NEBNext Ultra Directional RNA Library Prep Kit) according to instructions of the manufacturer. Whole transcriptome sequencing data sequenced by HiSeq Sequencer, was filtered and mapped to Cattle genome (Btagenome Version 5.0.1 NCBI). HISAT2 was used to calculate the gene count of mRNA and lncRNA.

### DEG Analysis and ceRNA Relation Prediction

Differentially expressed genes (including mRNA and lncRNA) analysis was applied utilizing DESeq under following criteria: Fold Change>2; FDR<0.05 23. We introduced the Miranda package to predict miRNA target on the 3'UTR region of differentially expressed mRNA as well as the full length sequence of differentially expressed lncRNA sequence. ceRNA relations were constructed by a pair of lncRNA and mRNA affected by the same miRNA members.

### Co-Expression network analysis

Gene co-expression networks were employed to demonstrate the interrelations among lncRNA and genes. They were established on the basis of the normalized expression values of genes. The genes were chosen in terms of the significant GO-terms and pathway-terms as well as differentially expressed lncRNA 24. In order to construct the networks, genes were paired and the Pearson Correlation of each pair was used to determine the pairs of significant correlation (FDR<0.05). k-cores in graph theory were employed to make the graph topology analysis simplified, which could represent the core status of a lncRNA among genes and lncRNA.

### Statistical analysis

All the data were analyzed by SPSS17.0 statistical software (SPSS Inc., USA). Values are expressed as means ± standard deviation (SD). Two-tailed Student's t-test as well as one-way ANOVA were used to statistically analyze the difference between two or multiple groups, respectively. When a *p*-value was less than 0.05, the group difference was thought to be statistically significant.

## Results

### Spontaneous conversion of human skin FBs into KLCs in a time-dependent manner

**Figure 1:**
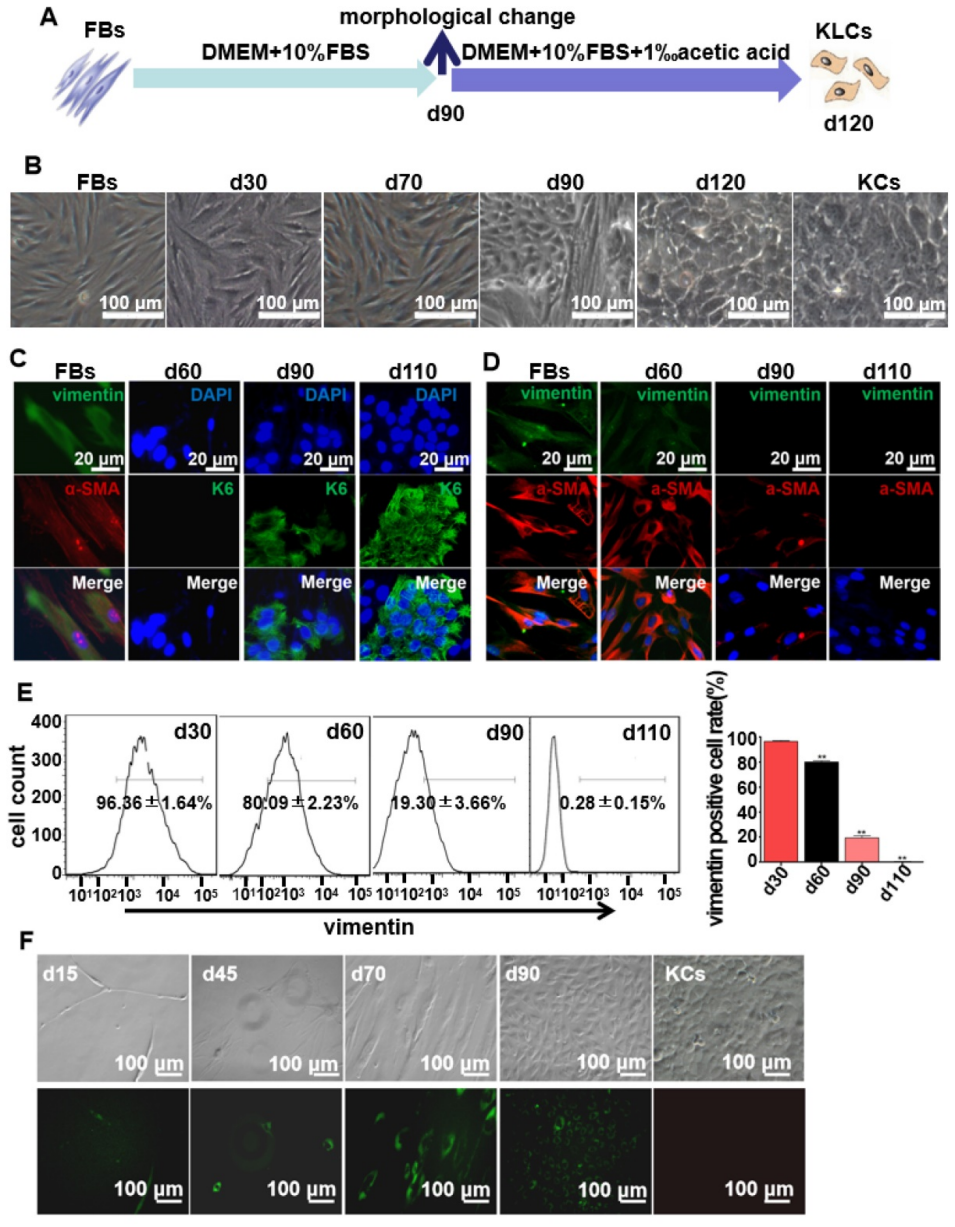
** Spontaneous conversion of human skin FBs into KLCs.** (A) Schematic of the conversion from FBs towards KLCs. (B) Representative bright field pictures during the course of time-dependent natural conversion. (C) Immunofluorescence labeling assays showing the expression of the indicated markers for FBs (vimentin and α-SMA) and keratinocytes (KCs) (keratin 6 (K6)) during the course of cell-fate conversion. (D) Immunofluorescence labeling assays showing the cell-fate conversion with FBs markers (vimentin and α-SMA). (E) Flow cytometry analysis of FBs marker (vimentin) over the conversion course on FBs. **P<0.01, n=5 in each group. (F) The conversion course of GFP^+^ FBs screened with G418 showing that the FBs started to become converted into KLCs on day 90. Top row: bright field images; Bottom row: the corresponding fluorescence images. The first four panels in F are cells cultured for 15, 45, 70 and 90 days, respectively. The last panel in F is KCs isolated from human skin but without GFP transfection, showing that the FBs-derived KLCs were morphologically similar to KCs.

FBs were isolated from human foreskins and then routinely cultured for more than 120 days in regular culture medium (Figure 1A). Without the addition of special inducers as in the previously reported cell reprogramming method 25, the long-term culture enabled us to discover the unexpected spontaneous conversion of FBs into KLCs over the time (Figure 1B and S1A). In the control experiments, three kinds of FBs isolated from adipocyte tissues, tumors, and hypertrophic scars could not be converted into KLCs on day 120 (Figure S1B). Immunostaining assays showed that FBs were converted to KLCs, which were positive in the protein K6 26, in a time-dependent manner (Figure 1C and S2A). The number of FBs (vimentin and α-SMA positive cells 27, 28) started to decrease from day 60 post primary cells culture, and by day 110 vimentin or α-SMA positive cells could not be detected any more (Figure 1D and S2B). To quantify the conversion of the cell types, vimentin positive cells were evaluated by flow cytometry. The percentage of vimentin positive cells was 96.36±1.64%, 80.09±2.23%, 19.30±3.66% and 0.28±0.15% on day 30, 60, 90 and 110, respectively, with a sharp declining on day 90 (Figure 1E, P<0.01, n=5 in each group). Namely, the number of FBs was decreasing while that of the KLCs was increasing due to the FBs-KLCs conversion over the time, and the significant conversion occurred on day 90.

In order to exclude the possibility that KLCs were converted from the epibiotic KCs of human skin originally mixed with FBs from epidermal layer, we transfected GFP^+^ plasmids into the primary FBs and utilized G418 to screen GFP^+^ FBs. We then tested whether the screened GFP^+^ FBs could be spontaneously converted into KLCs. On day 15 and 45, only a small number of spindle-shaped FBs were observed and no KCs were found (Figure 1F). These FBs proliferated and were spontaneously converted into KLCs starting from day 90 (Figure. 1F). These results confirm that the KLCs were indeed generated by the conversion of FBs but not from the KCs present in the original culture of FBs.

### Characteristics of the converted KLCs

**Figure 2:**
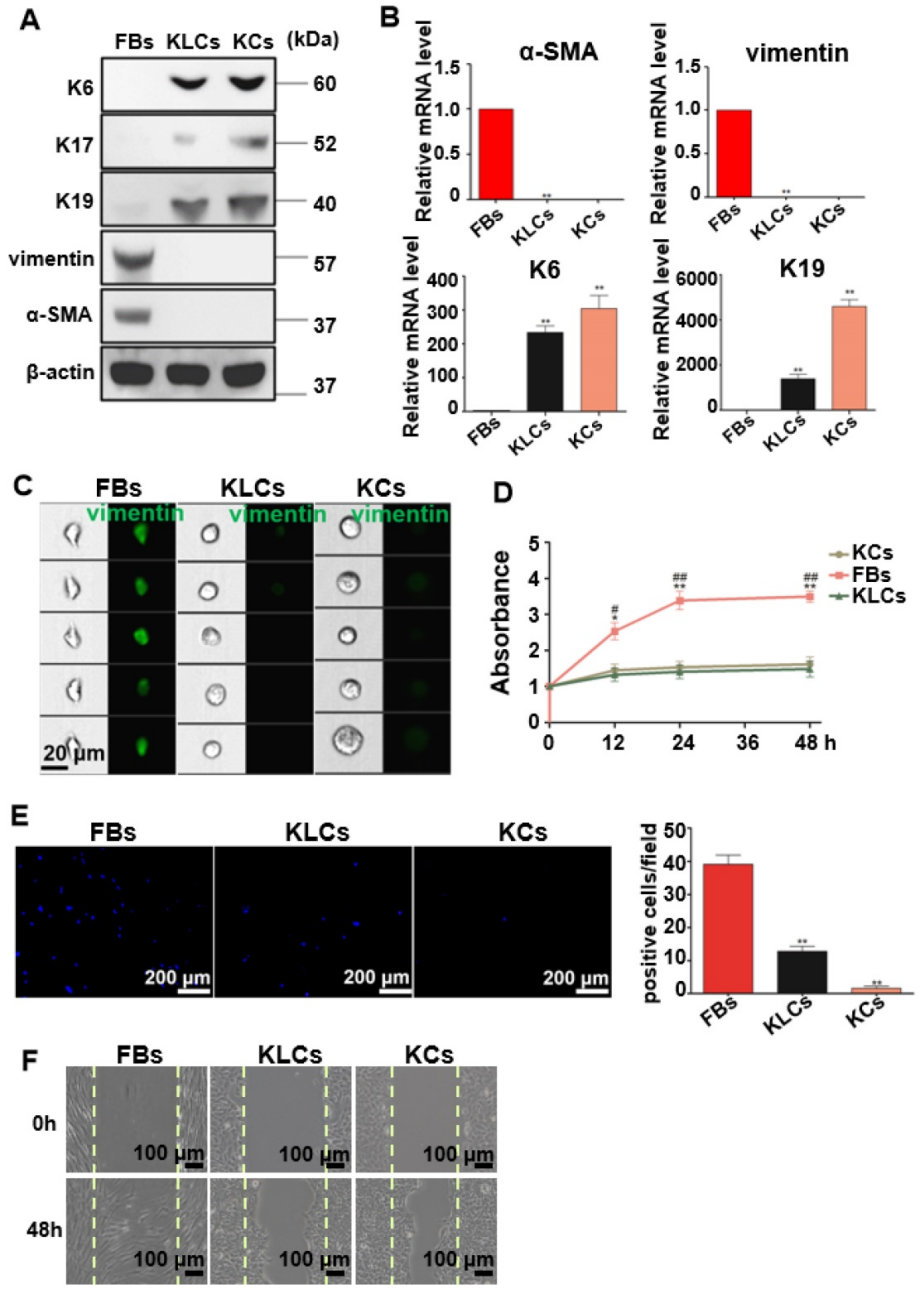
** Characteristics of the converted KLCs.** (A) Western blot results showing that KLCs highly expressed K6, K17 and K19 protein, but did not express FBs associated vimentin and α-SMA. (B) mRNA expression of α-SMA, vimentin, K6 and K19 by qRT-PCR. **P<0.01, n=5 in each group. (C) Image flow cytometry analysis exhibiting that FBs associated protein vimentin was not expressed in KLCs. KCs and KLCs were round diamond-shaped, which were significantly different from spindle-shaped FBs. (D) CCK8 assays showing the proliferation ability of FBs, KCs and KLCs. **P<0.01 and *P<0.05, the FBs group vs the KCs group, and ^##^P<0.01 and ^#^P<0.05, the FBs group vs the KLCs group, n=5 in each group. Cell migration was analyzed using Transwell assay (E), **P<0.01, n=5 in each group, and a wound scratch assay (F).

To well characterize the converted KLCs, we compared KLCs with FBs and KCs at the molecular and cellular levels. At the molecular level, Western blot assays revealed that KCs markers, including K6, K17 29 and K19 30, 31, were expressed in both KCs and KLCs, but were not expressed in FBs. However, FBs markers, including vimentin and α-SMA, were expressed in FBs, but were not expressed in KLCs or KCs (Figure 2A). Consistent with the Western blot results, qRT-PCR analysis confirmed that the level of mRNA expression of K6 and K19 in KLCs, though lower than that in KCs, was approximately 233 and 1,383 times higher than that in FBs, respectively. Moreover, a high level of mRNA expression of vimentin and α-SMA was detected in FBs and was over 1,000 times higher than that in KLCs (Figure 2B, P<0.01, n=5 in each group).

At the cellular level, image flow cytometry analysis exhibited that vimentin was positive in FBs, but not in KCs and KLCs (Figure 2C). Furthermore, KCs and KLCs were round diamond-shaped, significantly different from the spindle-shaped FBs (Figure 2C). CCK8 assays exhibited that the proliferation rate of FBs was significantly higher than that of KCs and KLCs at 12 h, 24 h and 48 h after cell seeding (Figure 2D, **P<0.01 and *P<0.05, the FBs group vs the KCs group, and ##P<0.01 and #P<0.05, the FBs group vs the KLCs group, n=5 in each group.) whereas there was no obvious difference between KCs and KLCs at each time point (Figure 2D, P>0.05, n=5 in each group). Transwell assays further showed that the number of migrated cells was decreased in the order of the FBs group, the KLCs group and the KCs group (Figure 2E, P<0.01, n=5 in each group). The number of FBs undergoing migration in the wound scratch assay was similar to that of KCs and KLCs (Figure 2F). These results suggest that the proliferation ability of KCs and KLCs was similar but significantly lower than that of FBs, confirming the need to generate KLCs from FBs.

### KLCs converted from FBs accelerate wound healing

To determine the therapeutic effects of converted KLCs on wound healing, full-thickness skin wounds were created on the back of nude mice, treated with saline, FBs, KLCs, or KCs, and then examined on day 6,12, 18 and 28 post wounding (Figure S3A upper left panel). Gross observation revealed that the wound-healing rate on day 12, 18 and 28 was 86.80±3.96%, 93.40±2.07%, and 99.80±0.45%, respectively, in the KLCs group. The wound treated with KLCs was closed significantly sooner than that treated with saline (Figure S3A upper right panel, 38.20±2.59% on day 12, 57.60±4.28% on day 18 and 75.20±3.11% on day 28, P<0.01). On day 12 after wound was created, the wound-healing rate in the KLCs group was even higher than that in the KCs group (77.80±5.36%, P<0.05). Furthermore, on day 12 and 18 after wounding, the wound-healing rate in the KLCs group was also obviously higher than that treated with FBs (Figure S3A upper right panel, 74.80±2.86% on day 12, P<0.01, and 78.40±7.09% on day 18, P<0.05). On day 28, there was no significant difference among the groups of KLCs, KCs and FBs (Figure S3A, upper right panel, 99.80±0.45%, 99.60±0.55%, 98.40±1.14%, P>0.05 and lower representative pictures, n=5 in each group).

**Figure 3:**
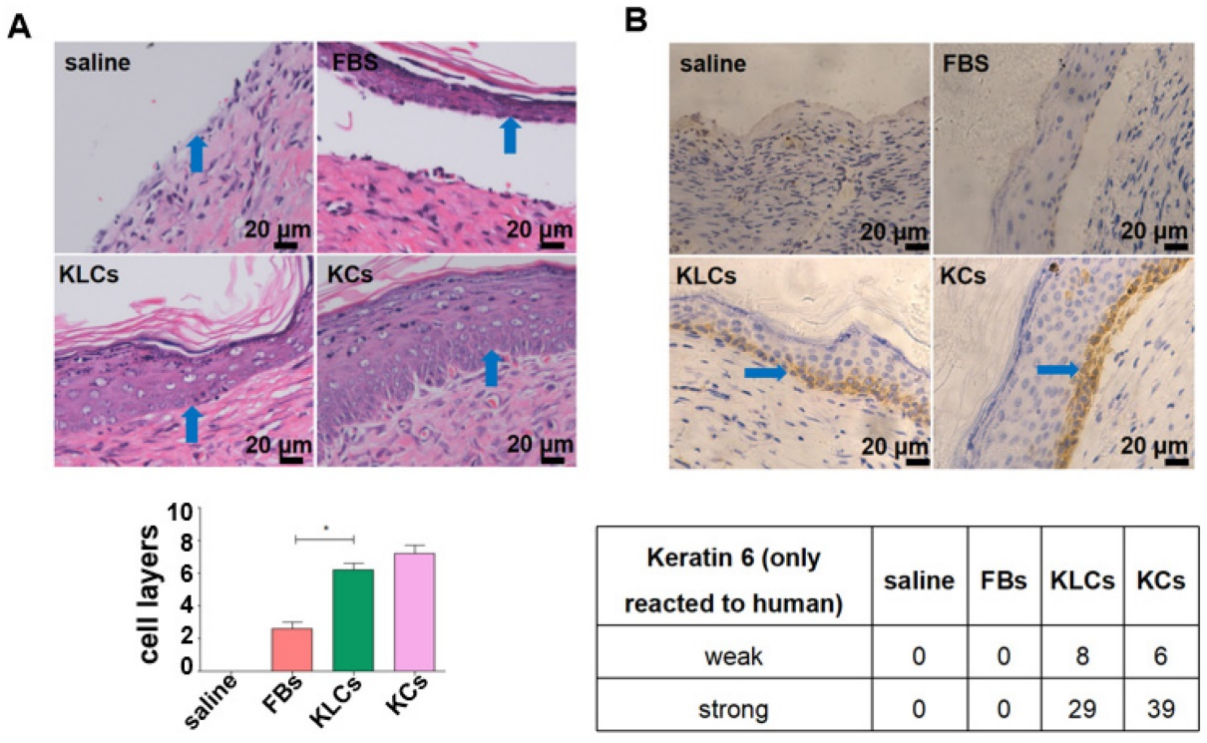
** Converted KLCs accelerated wound healing.** (A) H&E staining exhibiting that the epidermis in KLCs and KCs groups appeared to be much thicker and more adhesive than that in FBs group. *P<0.05, n=5 in each group. The areas indicated by the blue arrows was the interface between the epidermis and dermis. (B) Immunohistochemical staining showing the wound on the mice applied with KLCs contained K6 positive cells. The areas indicated by the blue arrows were the K6 positive cells.

Although there was no difference in the treatment effect among the FBs, KLCs and KCs groups on day 28 after wounding, the epidermis cell layers on the wound in the groups of KLCs and KCs were much thicker than those in the FBs group while there was no epidermis on the mouse wound with saline treatment (Figure 3A and S3B). Furthermore, the epidermis on the wound in the KLCs and KCs groups was more adhesive to the wound, which was closer to the physiological conditions, suggesting that the wound healing in the KLCs and KCs groups was much better than that in the FBs group (Figure 3A and S3B). In order to confirm that the KLCs were positively involved in the wound healing, K6 antibody (reactive to human antigen specifically) was used to immunohistochemically stain KLCs and KCs on the wound. The results showed that the FBs-derived KLCs and KCs, after transplanted, were reserved as a row of single cells between epidermis and dermis (Figure 3B and S3C), which played a “bridging” role in the wound healing.

### FBs-converted KLCs are of no tumorigenicity

**Figure 4:**
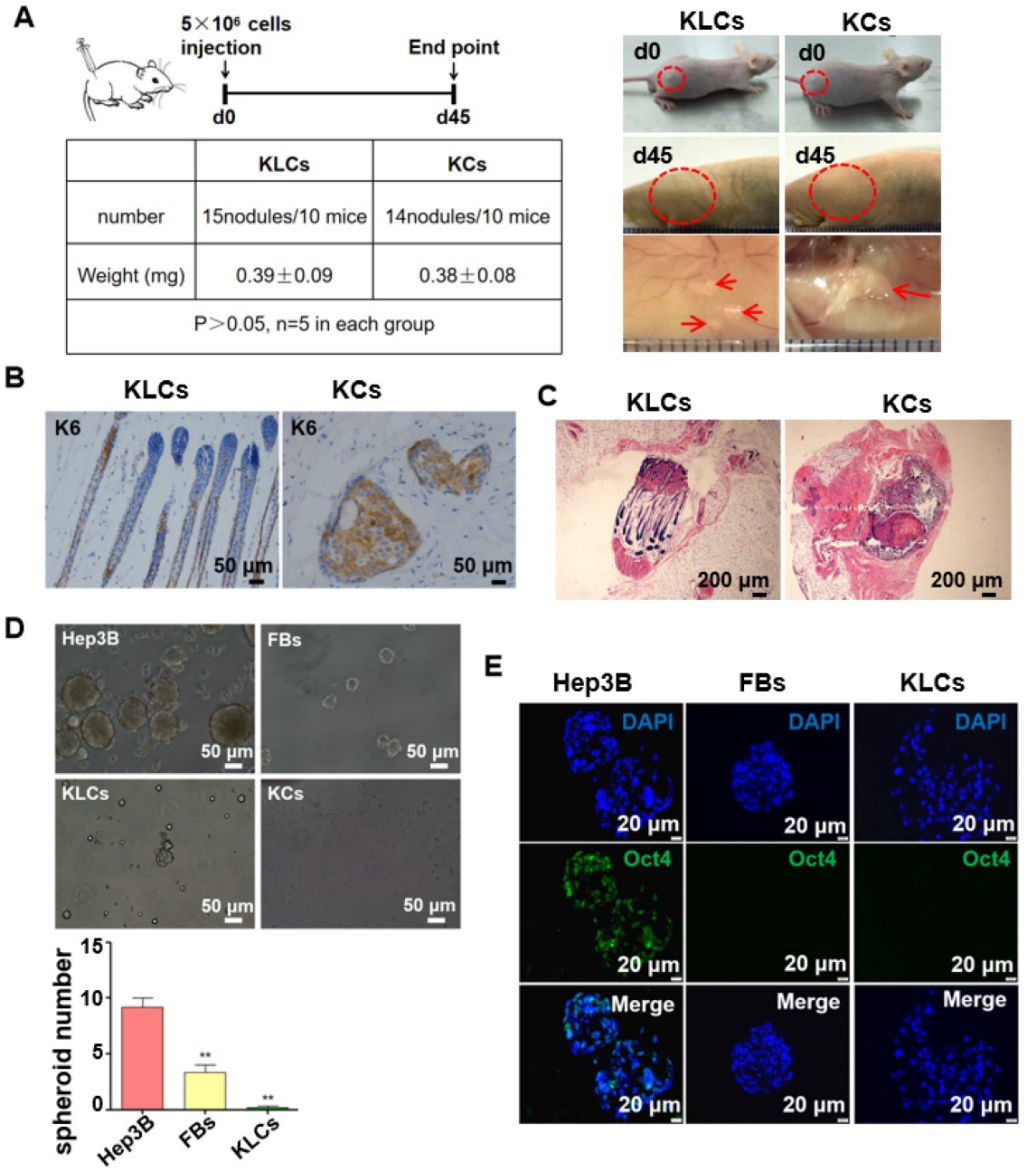
** Converted KLCs were safe in vivo and vitro.** (A) Tumorigenicity assay of KLCs and KCs on nude mice, n=5 in each group. The areas in the red circle were the cell injection sites on day 0 and day 45, and areas marked by red arrows were subcutaneous neoplasms 45 days after cell injection. (B) Immunohistochemical staining showing the neoplasms K6 positive cells in the KLCs and KCs groups. (C) H&E staining with the neoplasms in the KLCs and KCs groups. (D) Representative bright field pictures of spheroids culture. **P<0.01, n=5 in each group. (E) Immunofluorescence labeling assays exhibiting that the spheroids of Hep3B cells highly expressed Oct4, which was not expressed in the spheroids of KLCs or KCs.

The safety of the FBs-converted KLCs is significantly critical for their clinical application in burn wound healing. To test whether the KLCs have oncogenicity, a tumorigenicity assay was performed on the nude mice. The tumorigenicity assay showed no statistical difference in the subcutaneous neoplasms weight between both the KLCs and KCs groups 45 days after cell injection (Figure 4A, 0.39±0.09 mg vs. 0.38±0.08 mg, P>0.05, n=5 in each group). Immunostaining of the neoplasms using human K6 antibody suggested that there was no oncogenicity arising from KLCs in vivo (Figure 4B). Neoplasms in the KLCs groups showed a hair follicle structure by H&E staining (Figure 4C).

We cultured Hep3B cells (a tumor cell line as a control), FBs and KLCs to form spheroids on day 7 in vitro. We found that the spheroids derived from Hep3B cells were obviously larger than those from FBs and KLCs, and the spheroid number of Hep3B cells was much larger than other groups (Figure 4D, P<0.01, n=5 in each group). Immunofluorescence staining revealed that the positive rate of Oct4 (an indicator of tumor formation potential) was more than 95% in the Hep3B cells constituting the spheroids, but Oct4 was not expressed in KLCs and FBs constituting the respective spheroids (Figure 4E). These results indicated that both KLCs and FBs did not have a potential of inducing tumor formation and thus are safe for in vivo application.

### AKT3 is identified as a central element during epidermal conversion from FBs

**Figure 5:**
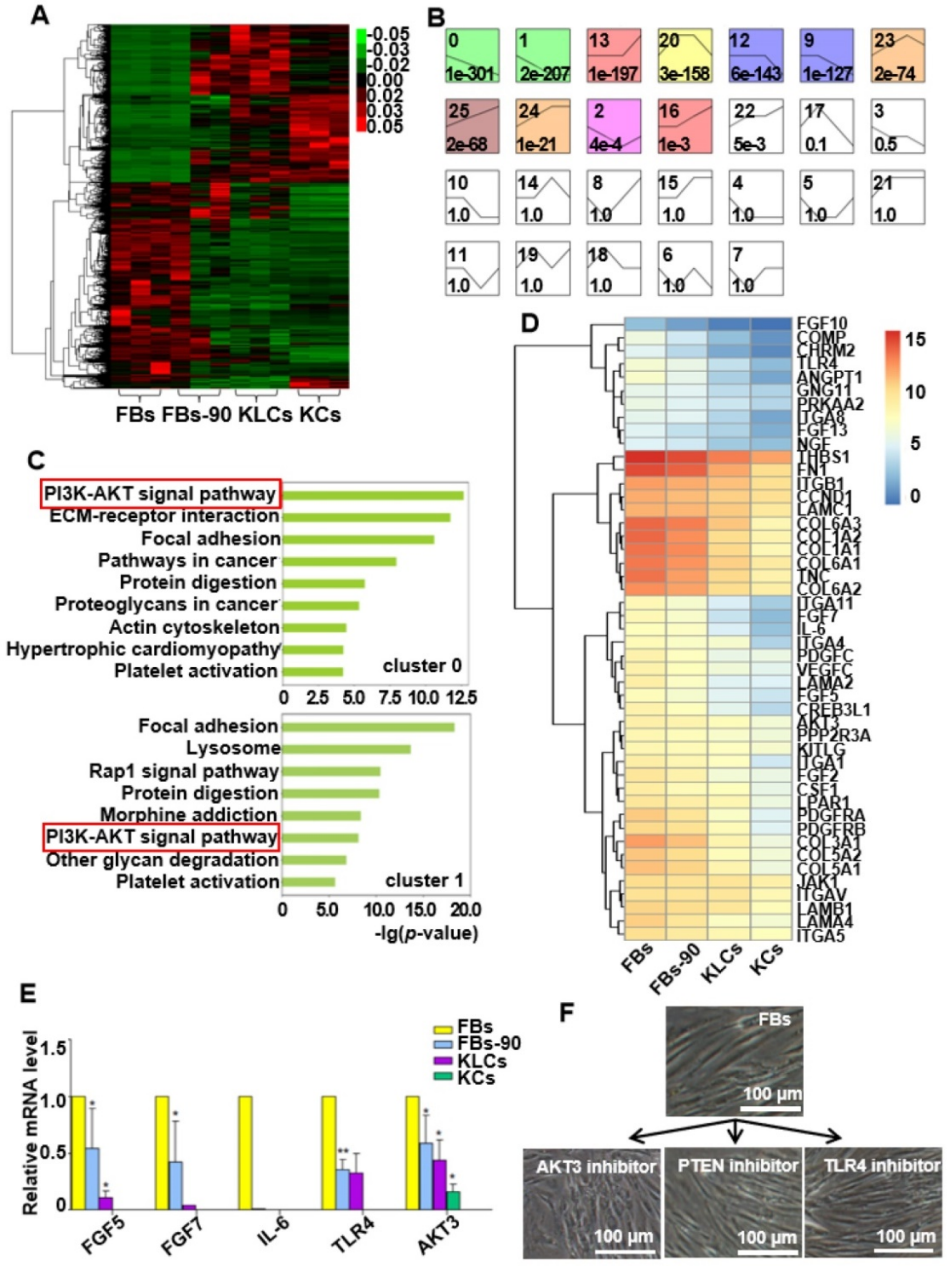
** AKT3 was identified as a central element during epidermal conversion from FBs.** (A) Heat map of differentially expressed genes determined by performing RNA-microarray analysis on FBs, FBs-90, KLCs, KCs. (B) 26 clusters were divided from the mRNA trend on the basis of the gene expression patterns of the four cell stages. The cluster number was indicated on the top left corner whereas the *p*-value significance of genes (assigned versus expected of each cluster) was denoted on the down left corner. (C) Pathway analysis for the overlapping genes among FBs, FBs-90, KLCs, KCs of clusters 0 and 1. (D) Heat-map of candidate genes with descending trend in the PI3K-AKT signal pathway. (E) qRT-PCR analysis of the five top candidate genes according to results from high-throughput sequencing. *P<0.05, n=5 in each group. (F) The effect of AKT3, PTEN and TLR4 inhibitors on epidermal conversion from FBs cultured for 14 days.

To identify candidate mRNAs that promoted the epidermal cellular conversion, mRNA profiles were firstly analyzed via high-throughput RNA sequencing in FBs, FBs cultured for 90 days (termed FBs-90), KLCs and KCs. A heat map of mRNAs was generated and distinguished the four cell types. The heat map showed a dynamic and continuous evolution in the expression profiles from FBs, to FBs-90 and finally to KLCs (Figure 5A). It demonstrated that the mRNA expression profiles were distinct between KLCs and FBs, but were much alike between KLCs and KCs. In order to disclose the underlying mechanisms involved in the epidermal cellular conversion, the markedly changed mRNAs were subsequently analyzed according to their dynamic expression patterns and 26 clusters were depicted (Figure S4). Among them, 11 colored clusters indicated that significantly enriched mRNAs were detected with statistical significance. Number 0 and 1 with down-regulated expression trend and number 16, 24 and 25 with up-regulated expression trend were selected for the subsequent study. When the files of these clusters were ordered based on the *p*-value significance of genes assigned, number 0 and 1 clusters had a much lower *p*-value than the other clusters (Figure 5B). Accordingly, these two clusters were considered as the focus of our following research.

We applied a pathway analysis on number 0 and 1 clusters to categorize the altered genes that affected the epidermal cellular conversion. The pathway analysis exhibited that the PI3K-AKT signal pathway was highly enriched in genes of clusters 0 and 1 (Figure 5C). The 46 mRNAs with a descending trend enriched in the PI3K-AKT signal pathway were picked out as the result of high-throughput sequencing (Figure 5D). Of the 46 annotated mRNAs, FGF5, FGF7, IL-6, TLR4 and AKT3 were located mainly in the middle and upper regions of the PI3K-AKT pathway (Figure S5) and thus drew our attention. qRT-PCR analysis disclosed that the expression of the five candidate genes were decreased over the process of epidermal conversion (Figure 5E, *P<0.05, **P<0.01, n=5 in each group), which further verified the results of high-throughput sequencing.

AKT3 is one of the most important molecules in the PI3K-AKT pathway. Hence, to further confirm the AKT3 effect on the FBs-KLCs conversion, inhibitors of AKT3, TLR4 and PTEN were added into the culture medium of FBs to suppress the expression of AKT3, TLR4 and PTEN, respectively. TLR4 and PTEN were used as a negative control because they did not show a trend of descending expression and were not involved in the FBs-KLCs conversion. Surprisingly, FBs treated with the AKT3 inhibitor were converted to polygonal KLCs after 14 days, but such conversion did not occur in the FBs treated with either the TLR4 or PTEN inhibitors (Figure 5F and Figure S6A and S6B). This result indicated that the FBs-KLCs conversion followed PI3K-AKT pathway, and the down expression of AKT3 played a critical role in the epidermal conversion from FBs to KLCs.

### LncRNA LINC00672 expression is deceased during cell epidermal conversion

**Figure 6:**
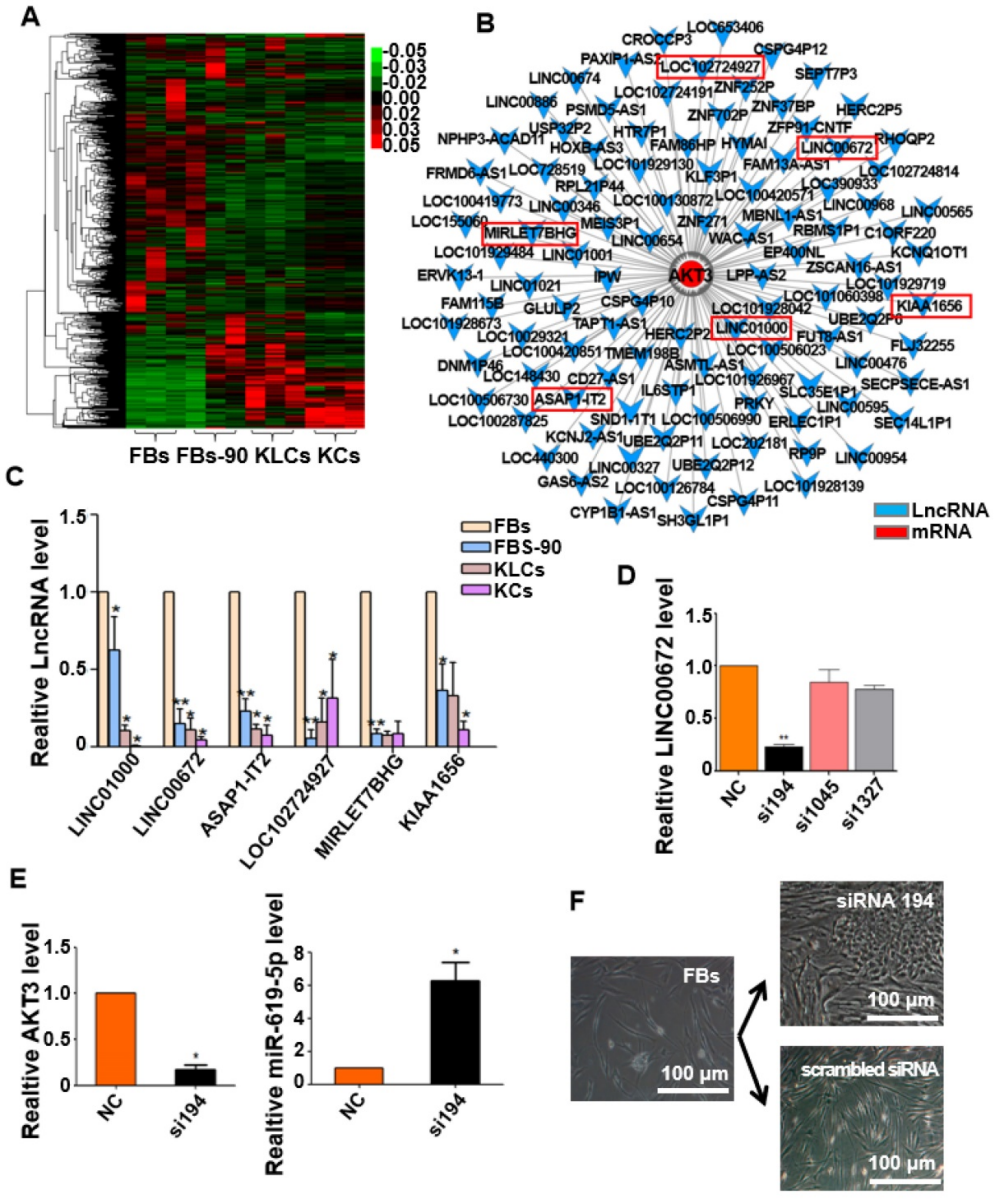
** Expression of LINC00672 was decreased during epidermal cellular conversion.** (A) Heat-map of differentially expressed genes determined by carrying out lncRNA-microarray analysis on FBs, FBs-90, KLCs, KCs. (B) The mRNA-LncRNA interacting network showing the predicted lncRNAs for the target mRNA AKT3. (C) qRT-PCR analysis of the six top candidate lncRNAs according to high-throughput sequencing in FBs, FBs-90, KLCs and KCs groups. *P<0.05, **P<0.01, n=5 in each group. (D) The relative LINC00672 expression in FBs inhibited with siRNA(si)194, si1045 and si1327 tested by qRT-PCR, and inhibitory effect of si194 was better than the other two siRNAs. **P<0.01, n=5 in each group. (E) Significant down-regulation of AKT3 level and up-regulation of miR-619-5p level due to the inhibition of si194. * p<0.05, n=5 in each group. (F) The inhibitory effect of si194 of LINC00672 on epidermal conversion from FBs cultured for 14 days.

It is well known that epigenetic regulation contributes much to cellular conversion 32, 33. LncRNAs have recently been in the spotlight as an important epigenetic regulatory factor 34, 35 and were then analyzed under our experimental setting. The heat map with differentially expressed lncRNAs clearly distinguished the four cell states (FBs, FBs-90, KLCs and KCs). lncRNAs expression pattern was different between FBs and FBs-90 whereas KLCs had a similar lncRNAs expression pattern as KCs (Figure 6A). In order to demonstrate the reason for AKT3 declining found during the process of cellular conversion (Figure 5D), an AKT3-lncRNA interacting network was drawn from the high-throughput sequencing of both mRNAs and lncRNAs (Figure 6B). The top 6 down-regulated lncRNAs targeting AKT3 were picked out (Figure 6B, Table S1). qRT-PCR analysis showed that the expression trends of LINC00672, LINC01000, ASAP1-IT2 and KIAA1656 were in agreement with the high-throughput lncRNAs sequencing (Figure 6C). According to the value of energy and score in Table S1, LINC00672 was predicted as one of the most important regulators of AKT3. Then, relative expression of LINC00672 in FBs was inhibited with si194, si1045 and si1327. qRT-PCR showed that the inhibitory effect of si194 was better than the other two siRNAs and also down-regulated AKT3 expression significantly (Figure 6D, p<0.01, n=5 in each group, and Figure 6E, left panel, p<0.05, n=5 in each group).

**Figure 7:**
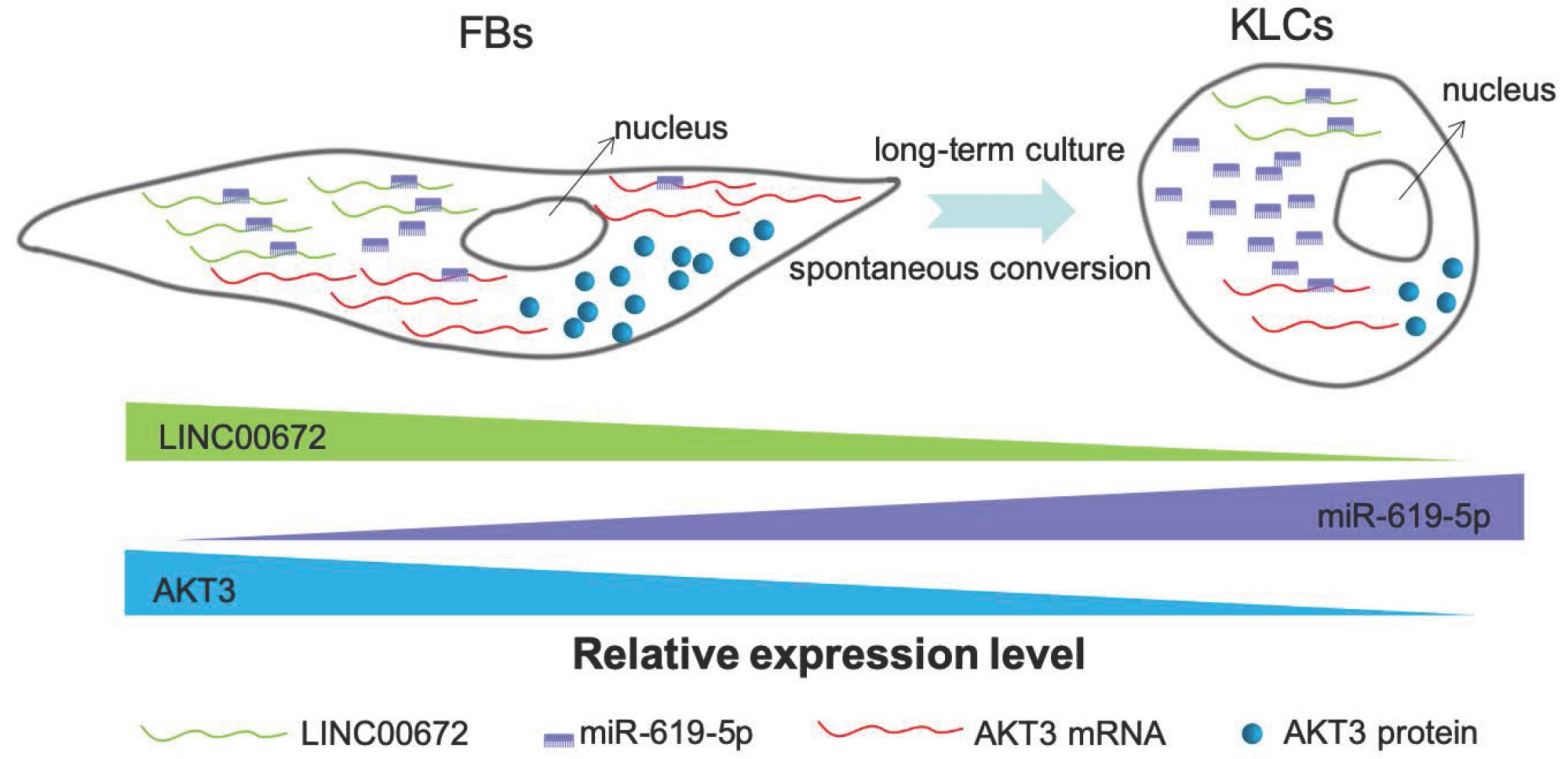
** The schematic diagram showing that LINC00672-mediated PI3K-AKT pathway regulated the FBs-KLCs conversion.** As FBs were spontaneously converted to KLCs, the number of LINC00672 in FBs was decreased, and their adsorption capacity for miR-619-5P was weakened, Consequently, more and more miR-619-5P molecules were combined with AKT3 mRNA, affecting its translation function.

Next we aimed to determine whether the AKT3 expression level in the epidermal cellular conversion was related to LINC00672. Miranda package was used to predict miRNA target on 3'UTR region of AKT3 and the full-length sequence of differentially expressed lncRNA sequence 36. miR-619-5p was then preferred as the key bridge between LINC00672 and AKT3 according to score and energy of ceRNA analysis (Table S2). qRT-PCR results revealed that LINC00672 suppression by si194 led to the down-regulation of AKT3 and up-regulation of miR-619-5p levels in FBs (Figure 6E, p<0.05, n=5 in each group). Meanwhile, qRT-PCR results exhibited that over-expression of LINC00672 in KLCs down- and up-regulated the level of miR-619-5p and AKT3, respectively (Figure S7A and S7B, p<0.05, n=5 in each group). Furthermore, FBs were treated with si194, 14 days later they were converted to polygonal KLCs, but such conversion was not found in the control group (Figure 6F and S7C). Collectively, all results exhibited that LINC00672 played a key role in the KLCs conversion, which could affect the expression of AKT3 by the absorption function of miR-619-5p (Figure 7 and S8).

## Discussion

Our study has revealed a spontaneous time-dependent conversion from human skin FBs into a functional KCs phenotype in vitro. Such conversion was significantly different from the previous studies 37. The global morphological change from FBs to KLCs was observed on day 90 in the primary culture. LINC00672-AKT3 interaction played an important role in epidermal cellular conversion, which could affect the expression of AKT3 by the absorption function of miR-619-5P. The absorption between LINC00672 and miR-619-5P occurred mainly relying on MRE 38, which formed an LINC00672 associated “miR-619-5P sponge” and affected mRNA expression of AKT3 (Figure 7 and Figure S8). The converted KLCs were as effective as KCs in healing the wounds, and were significantly more effective than the saline treatment. More importantly, the converted KLCs had no in vivo carcinogenicity and did not express pluripotent genes Oct4 in vitro, suggesting that the converted KLCs were safe.

Our work demonstrated that in vitro culture led to epigenetic modification to promote the conversion of FBs into KLCs, which served as the seed cells for skin regeneration. It is noteworthy that the spontaneous FBs-KLCs conversion has never been reported before although FBs have long been used as starting cells for generating iPSCs 39-41 and as feeder cells for ESCs in vitro 22, 42, 43. The spontaneous FBs-KLCs conversion could not be observed in these processes for three possible reasons. First, the culture time was usually short (less than one month) when FBs were used as cell sources for generating iPSCs or as feeder cells for culturing ESCs 22, 40. Second, when an effort is made to reprogram skin FBs into iPSCs by transfection of inductors, most FBs are not transfected and then die soon after screened culture. Third, when FBs are prepared as a feeder cell for culturing ESCs, FBs cultured for less than 5 generations are inactivated by mitomycin C 22.

So far, almost all the reported cellular conversion required the osmotic pressure shock, growth-factor deprivation, plasma membrane perforation, physical damage, heat shock, or transcriptional regulation 44, 45. Moreover, the cellular conversion rates reported were comparatively low, typically with an efficiency ranging from 0.085% to 3%, and required screening and culturing for one to two months 46, 47. Although it was reported that dermal FBs could be converted to KCs phenotype with a conversion rate of 53% only in 6 days, a combination of inducers (p63 and Klf4) needed to be delivered into dermal FBs and there were functional differences between KCs and induced KCs due to the incomplete reprogramming 48.

It should be noted that genetic manipulations raised safety concerns and were thus not desirable in most clinical applications 49, 50. Our method does not rely on genetic manipulation but still achieves time-dependent conversion of FBs into KLCs within ~110 days with a conversion rate of ~90% (Figure 1). Of note, when AKT3 in the PI3K-AKT pathway was inhibited, the conversion time was shortened from ~110 days to ~14 days (Figure 5F). Further, the KLCs produced by our method cannot induce tumor formation and are thus a safe cell source. What's more, it is well known that the proliferation efficiency of FBs is relatively fast in vitro. Hence, enough FBs can be easily obtained and then converted into enough KLCs in a short time. Generation of the safe and functional KLCs under our experimental settings hold great promise for repairing burning skins, as KCs are important seeding cells for tissue engineering.

Our present finding warned us that establishment of cell lines would lead to epigenetic modulation, which further results in changes in the characters and functions of the cells. In fact, this phenomenon is not uncommon. For example, anticancer drugs were normally screened by interacting with tumor cell lines cultured in vitro, but the in vitro cultured tumor cell lines did not fully represent the tumor cells in vivo due to the cell state anamorphose derived from long-term in vitro culture 51-53. Consequently, the anticancer drugs screened against in vitro cultured cells were not effective for treating tumors in vivo. Our work further suggested that the cells cultured in vitro for a long period of time could not be used to study the in vivo functions of the same cells 54.

In our study, although we identified that down-regulation of lncRNA-AKT3 under in vitro culture contributed much to FBs conversion into KLCs, we know very little about the underlying mechanisms, especially, why LINC00672 was down-regulated over the in vitro culture period. Another puzzling phenomenon is that only inhibiting LINC00672 (or AKT3) did not speed up the FBs-KLCs conversion although the level of this lncRNA molecule was found to decline over the in vitro culture. Hence, we believe LINC00672-mediated PI3K-AKT pathway regulated the FB-KLC conversion and other unknown genes (though not identified in this work) might be involved in the regulation of the epidermal cellular conversion.

## Conclusion

In summary, for the first time we discovered that human skin FBs could be spontaneously converted into KLCs in regular DMEM over a period of 110 days with a conversion rate of ~90%. We also found that the spontaneous conversion followed a LINC00672-mediated PI3K-AKT pathway. When key molecules such as AKT3 involved in this pathway was inhibited, the conversion time could be shortened from 110 days to 14 days. Suppressing the LINC0062 led to the downregulation of AKT3 in FBs, which promoted the spontaneous conversion. The KLCs, converted from FBs in this manner, were found to be effective in healing the wounds while not being oncogenic. Although KLCs did not proliferate rapidly, the rapid proliferation of FBs could enable the production of KLCs through the spontaneous conversion from FBs in a regular medium without special inducers. Hence, the discovered FBs-KLCs conversion opens a new revenue to the scalable production of cell sources needed to regenerate skins and healing wounds in large area.

## Supplementary Material

Supplementary figures and tables.

## Abbreviations

KCs: keratinocyte cells
FBs: fibroblasts
KLCs: keratinocyte-like cells
iPSCs: induced pluripotent stem cells
DMEM: Dulbecco`s Modified Eagle Media
PBS: phosphate-buffered saline
K: keratin
α-SMA: alpha smooth muscle actin
LncRNAs: Long non-coding RNAs
si: siRNA
MRE: miRNA response element
ESCs: embryonic stem cells
