# Supplementary material for: Spontaneous evolution of human skin fibroblasts into wound-healing keratinocyte-like cells
Source: Theranostics. 2019 Jul 9;9(18):5200–13. doi: 10.7150/thno.31526 (PMC6691578; doi:10.7150/thno.31526)
Supplement: Supplementary file 1 — Supplementary figures and tables. [file thnov09p5200s1.pdf]

Supplementary Figure Legend

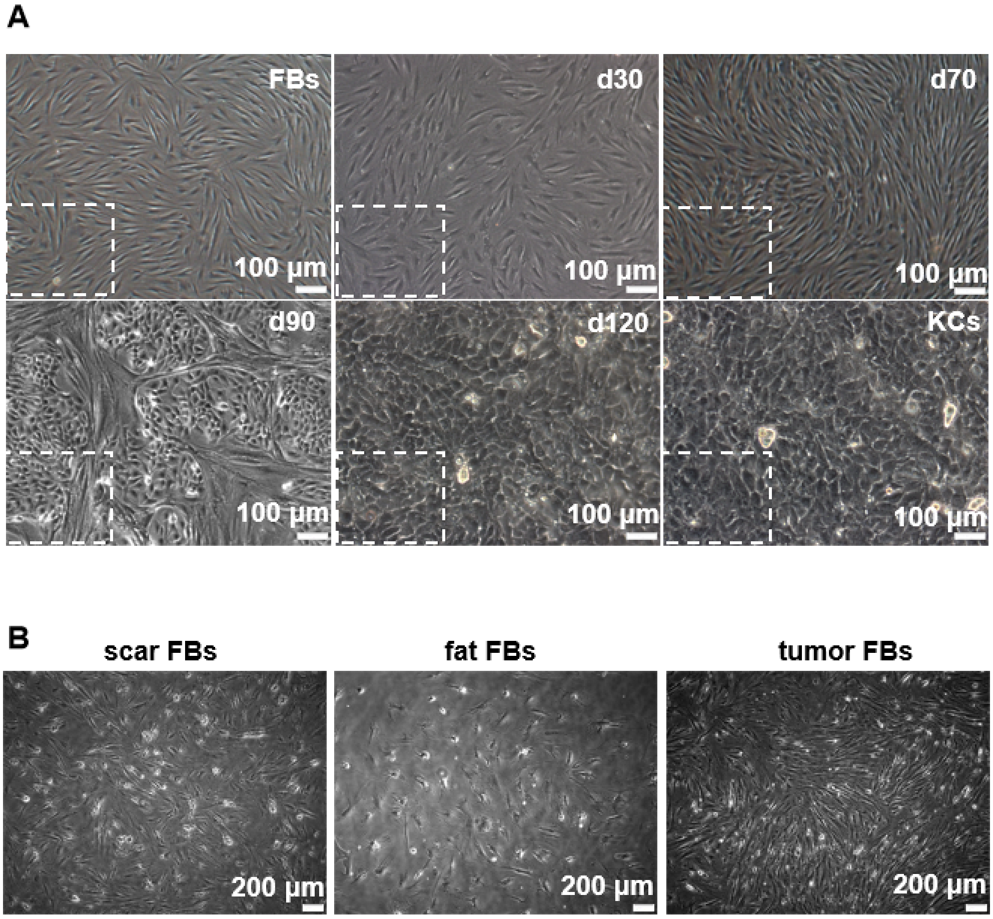

Supplementary Figure 1. Representative bright field pictures of natural conversion.

(A) Representative bright field pictures during the course of time-dependent natural conversion. High-magnification images of the areas framed with white dotted lines were shown in Fig. 1B. (B) Representative bright field pictures of scar FBs, fat FBs and tumor FBs cultured on day 120.

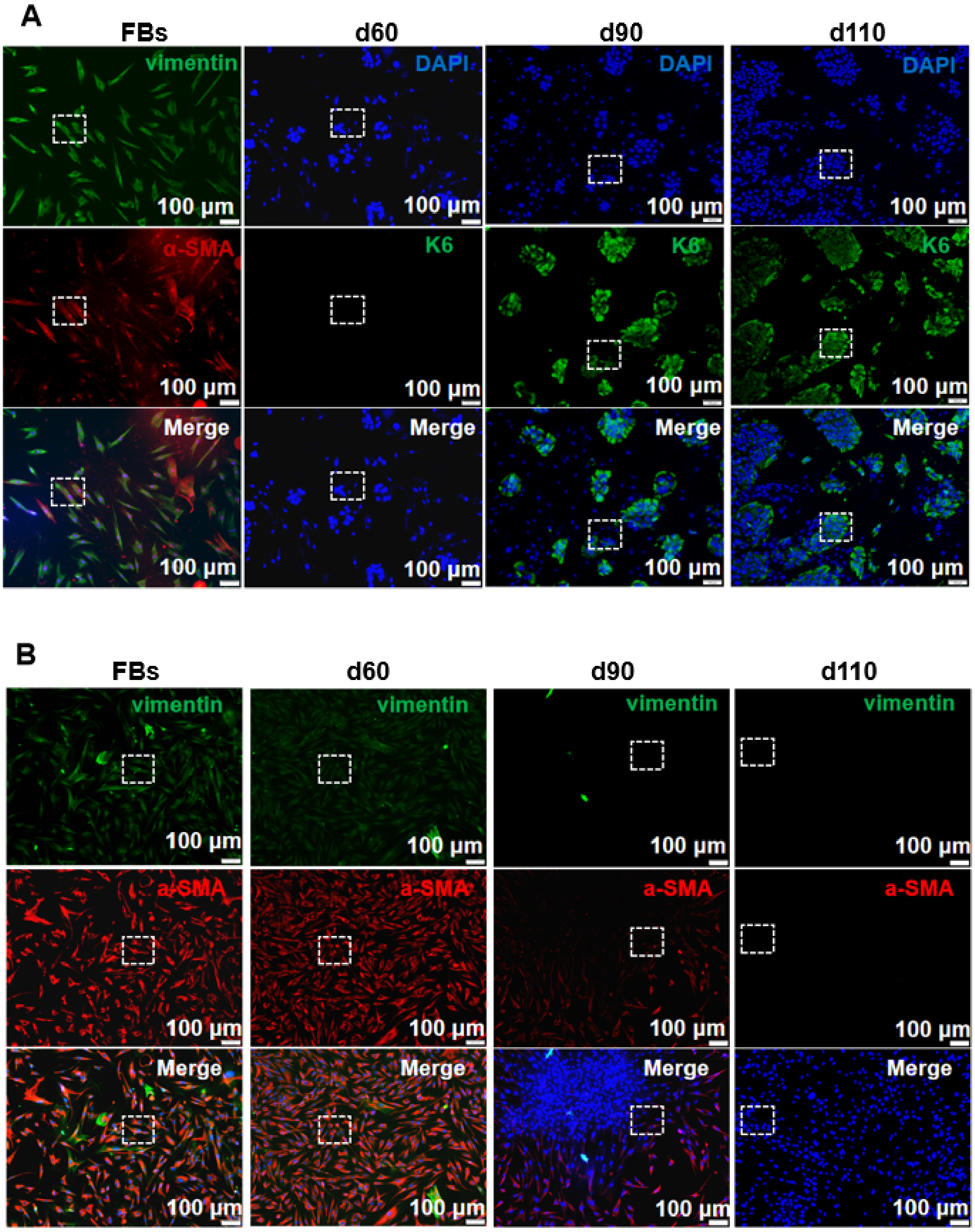

Supplementary Figure 2. Immunofluorescence labeling assays showed the expression of vimentin, α-SMA and keratin 6.

(A) Immunofluorescence labeling assays showed the expression of indicated FBs markers (vimentin and α-SMA) and keratinocytes (KCs) marker (keratin 6 (K6) during the course of cell-fate conversion. High-magnification images of the areas framed with white dotted lines were shown in Fig. 1C. (B) Immunofluorescence

labeling assays showed cell-fate conversion with FBs markers (vimentin and α-SMA). High-magnification images of the areas framed with white dotted lines were shown in Fig. 1D.

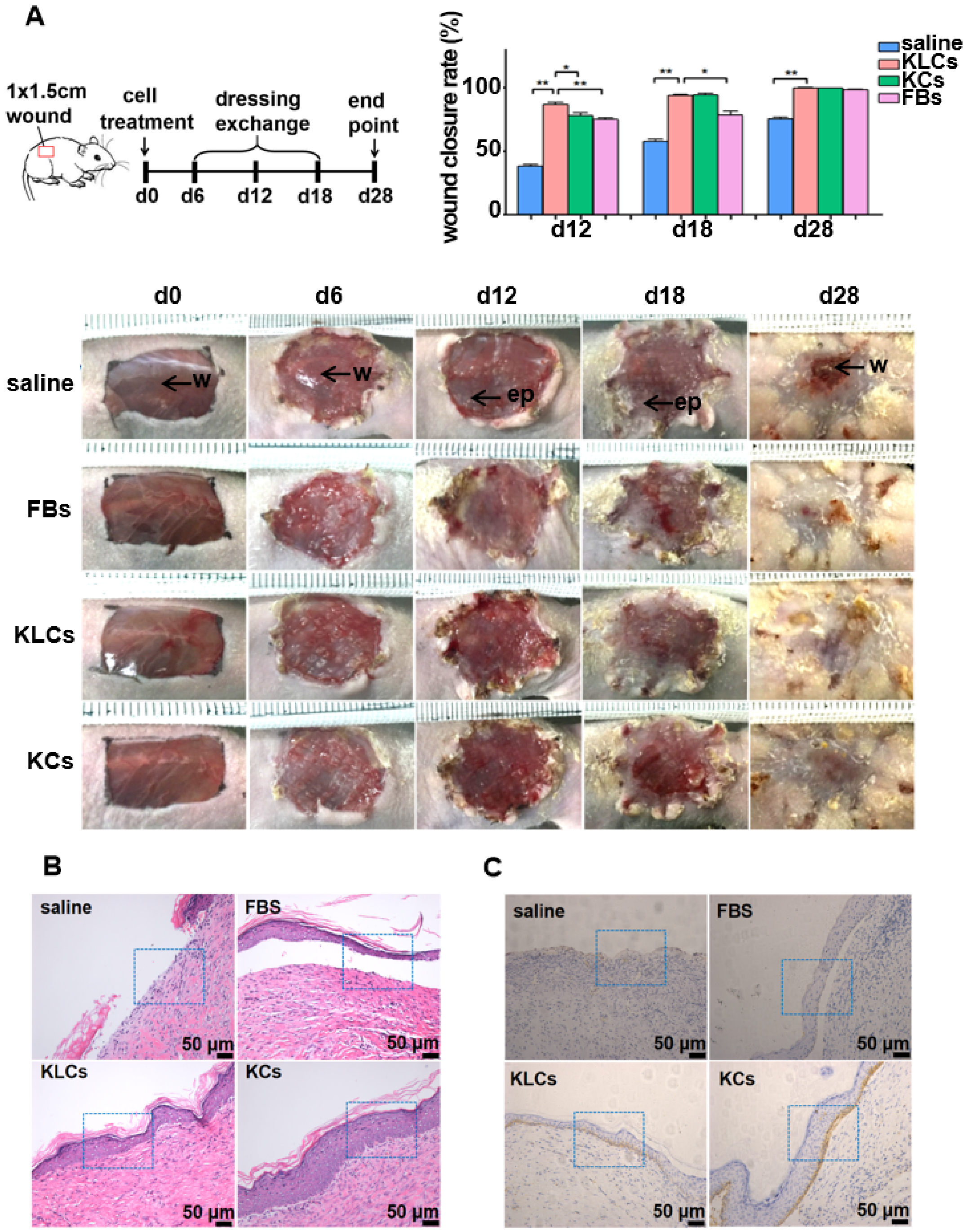

Supplementary Figure 3. Converted KLCs accelerated wound healing.

(A) Gross observation and statistical data showed that wound healing was sooner in

the KLCs group on day 12 and 18 post-wounding, compared with FBs and saline groups. The wound healing was also obviously sooner than the saline group on day 28 post-wounding. Arrowheads mark w as wound and ep as epithelization. *P<0.05 and **P<0.01, n=5 in each group. (B) H&E staining exhibited that the epidermis in KLCs and KCs groups appeared to be much thicker and more adhesive than that in FBs group. High-magnification images of the areas framed with blue dotted lines were shown in Fig. 3A. (C) Immunohistochemical staining showed the wound on the mice applied with KLCs contained K6 positive cells. High-magnification images of the areas framed with blue dotted lines were shown in Fig. 3B.

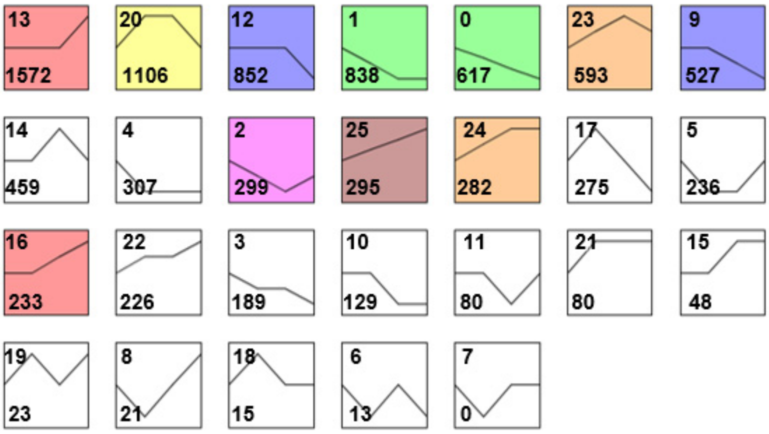

Supplementary Figure 4. Clusters were classified by the trend of mRNAs on the basis of the dynamic expression patterns.

26 clusters were classified by the trend of mRNAs on the basis of the dynamic expression patterns of FBs, FBs-90, KLCs and KCs. The cluster number was indicated on the top left corner and number of mRNAs assigned in each cluster was

denoted on the down left corner. 11 colored clusters indicated significantly enriched detected mRNAs with statistical significance.

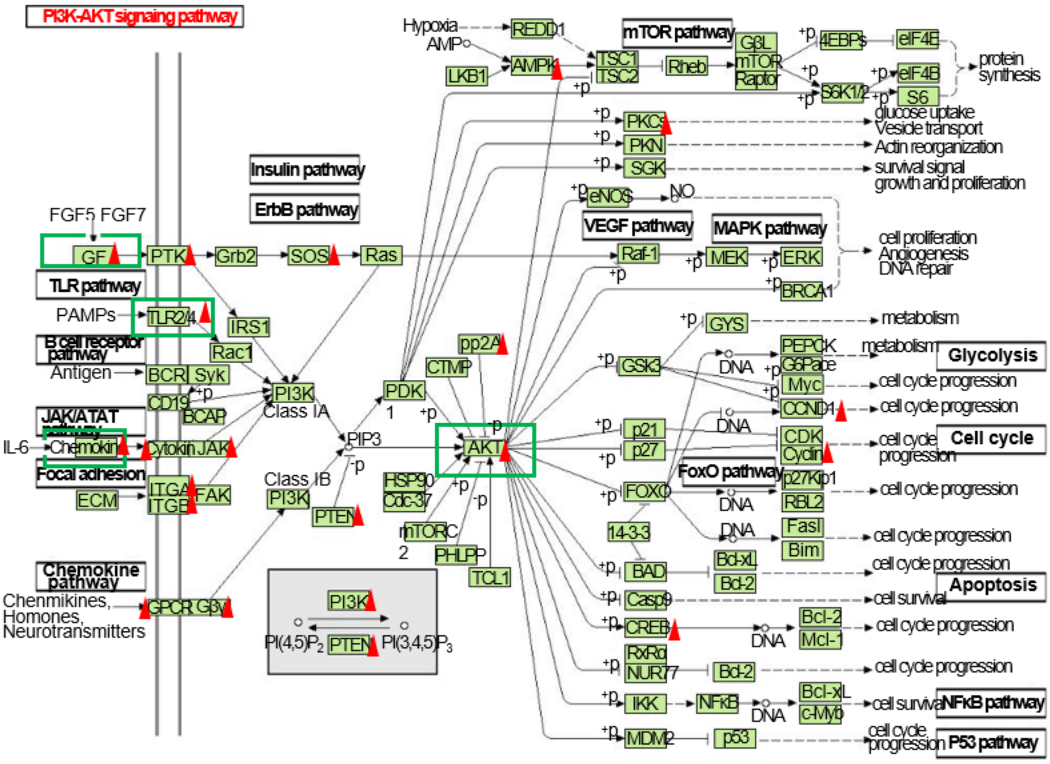

Supplementary Figure 5. The pathway diagram of PI3K-AKT.

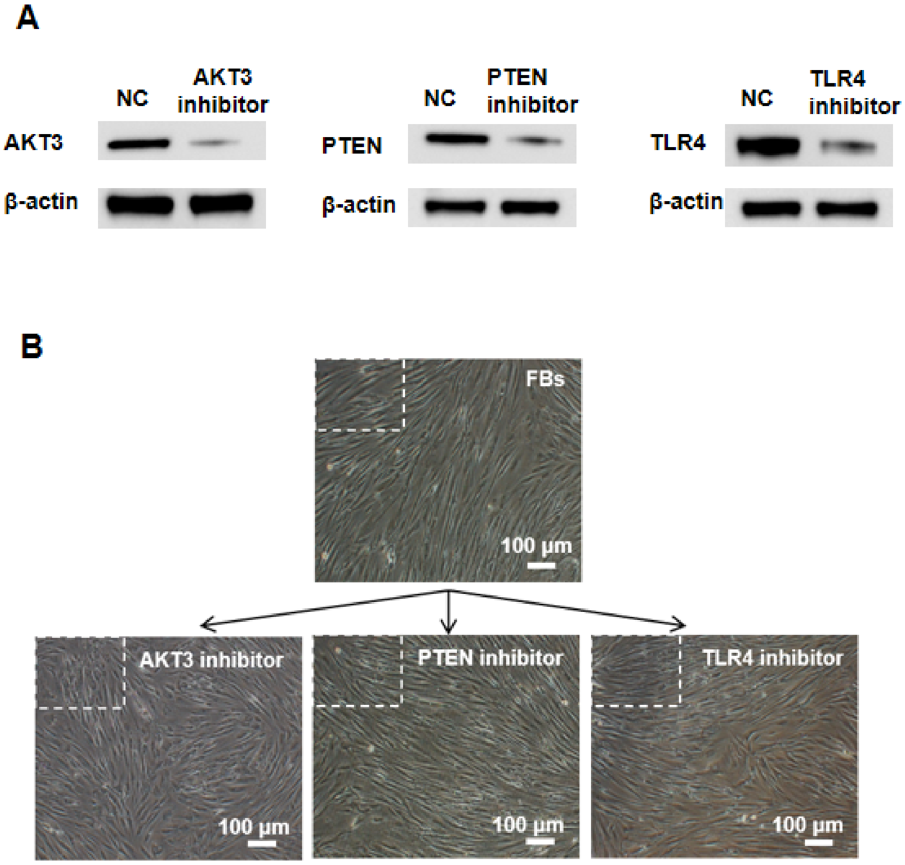

Supplementary Figure 6. Representative pictures on the epidermal conversion.

(A) Western blot results showing the effect of AKT3, PTEN and TLR4 inhibitors on FBs. (B) High-magnification images of the areas framed with white dotted lines were shown in Fig. 5F.

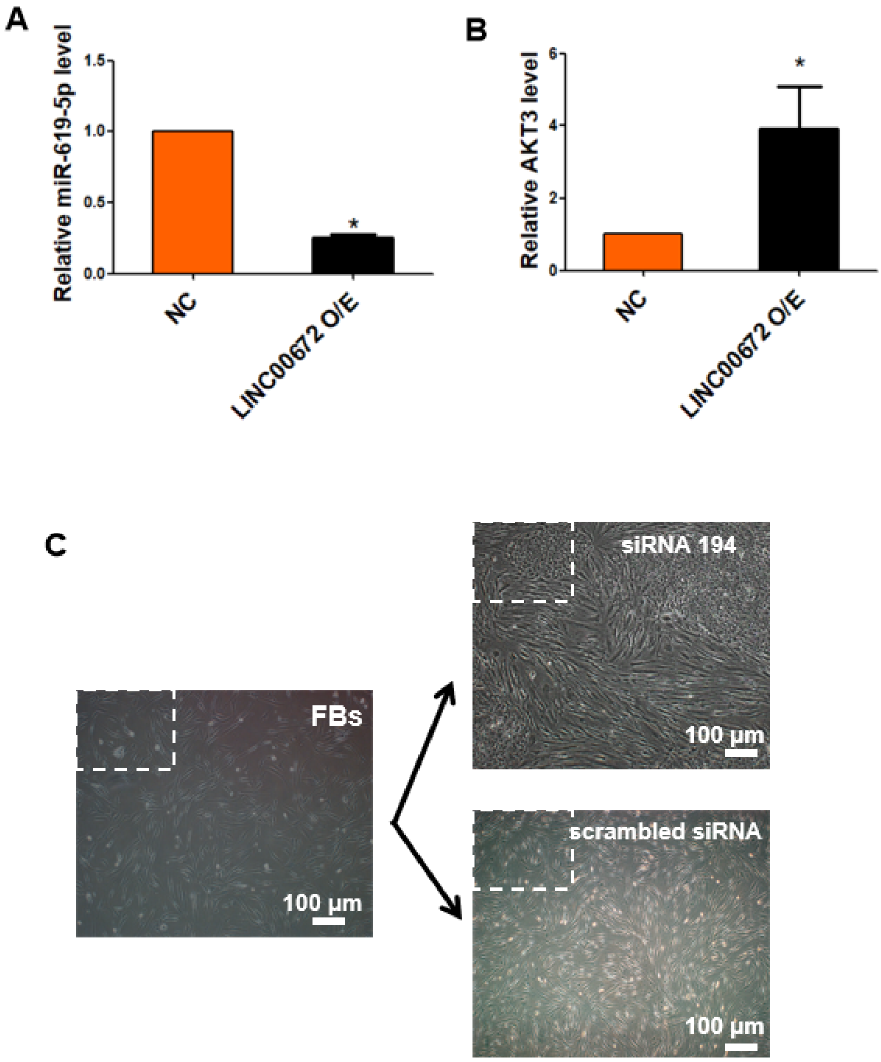

Supplementary Figure 7. LINC00672 involved in epidermal conversion.

(A) Significant down-regulation of miR-619-5p by overexpressed LINC00672 in KLCs. * p<0.05, t-test, n=5 in each group. (B) Significant up-regulation of AKT3 level by overexpressed LINC00672 in KLCs. * p<0.05, n=5 in each group. (C) The inhibitory effect of si194 on epidermal conversion from FBs cultured for 14 days. High-magnification images of the areas framed with white dotted lines were shown in Fig. 6F.

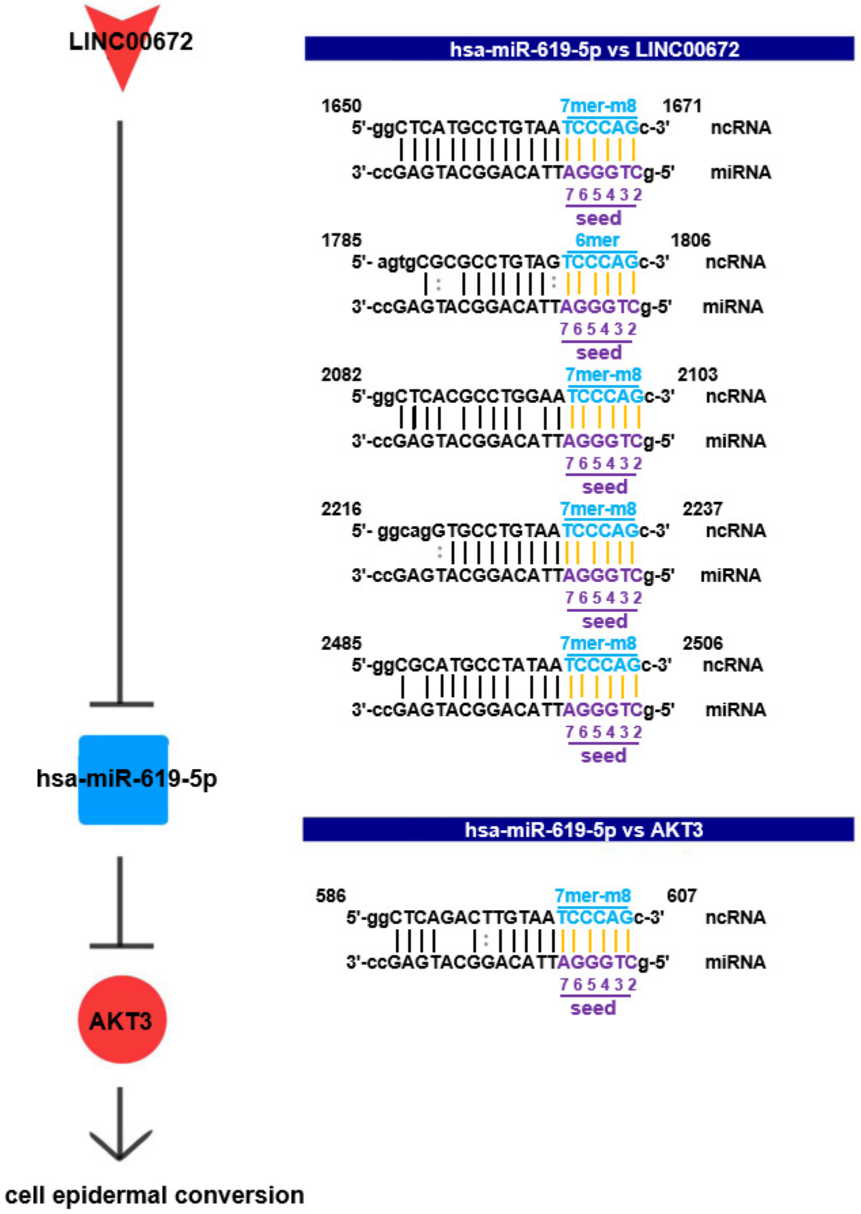

Supplementary Figure 8. The path diagram showing LINC00672-mediated PI3K-AKT pathway regulated the FBs-KLCs conversion.

Supplementary Table Legend

Supplementary Table 1. Top 6 down-regulated lncRNAs targeting to AKT3.

AccID	Energy	Score	StartSubject	Endsubject
LINC00672	-47.05	200	1650	1671
LOC102724927	-47.05	192	1622	1643
ASAP1-IT2	-42.14	180	690	711
MIRLET7BHG	-47.05	176	2160	2181
KIAA1656	-32.41	200	5333	5353
LINC01000	-42.14	177	4791	4812

Supplementary Table 2. ceRNA analysis of AKT3 and target lncRNAs

mRNA_SeriesCluster			mRNA_miRNA						lncRNA_miRNA						lncRNA_Series Cluster		
AccID	Profile	type_of_gene	QueryID	SubjectID	Energy	Score	StartSubject	Endsubject	QueryID	SubjectID	Energy	Score	StartSubject	Endsubject	AccID	Profile	type_of_gene
AKT3	9	protein-coding	hsa-miR-619-5p	AKT3	-36.84	180	586	607	hsa-miR-619-5p	KIAA1656	-42.14	192	1173	1194	KIAA1656	1	ncRNA
AKT3	9	protein-coding	hsa-miR-619-5p	AKT3	-36.84	180	586	607	hsa-miR-619-5p	KIAA1656	-32.41	162	5333	5353	KIAA1656	1	ncRNA
AKT3	9	protein-coding	hsa-miR-619-5p	AKT3	-36.84	180	586	607	hsa-miR-619-5p	LINC00672	-47.05	200	1650	1671	LINC00672	0	ncRNA
AKT3	9	protein-coding	hsa-miR-619-5p	AKT3	-36.84	180	586	607	hsa-miR-619-5p	LINC00672	-37.51	184	2082	2103	LINC00672	0	ncRNA
AKT3	9	protein-coding	hsa-miR-619-5p	AKT3	-36.84	180	586	607	hsa-miR-619-5p	LINC00672	-35.9	184	2485	2506	LINC00672	0	ncRNA
AKT3	9	protein-coding	hsa-miR-619-5p	AKT3	-36.84	180	586	607	hsa-miR-619-5p	LINC00672	-38.35	181	2216	2237	LINC00672	0	ncRNA
AKT3	9	protein-coding	hsa-miR-619-5p	AKT3	-36.84	180	586	607	hsa-miR-619-5p	LINC00672	-34.23	162	1785	1806	LINC00672	0	ncRNA
AKT3	9	protein-coding	hsa-miR-619-5p	AKT3	-36.84	180	586	607	hsa-miR-619-5p	LPP-AS2	-32.79	175	2025	2046	LPP-AS2	1	ncRNA
AKT3	9	protein-coding	hsa-miR-619-5p	AKT3	-36.84	180	586	607	hsa-miR-619-5p	LPP-AS2	-33.35	164	1890	1911	LPP-AS2	1	ncRNA
AKT3	9	protein-coding	hsa-miR-619-5p	AKT3	-36.84	180	586	607	hsa-miR-619-5p	RP9P	-42.14	192	331	352	RP9P	0	pseudo
AKT3	9	protein-coding	hsa-miR-619-5p	AKT3	-36.84	180	586	607	hsa-miR-619-5p	LOC102724927	-47.05	200	1622	1643	LOC102724927	1	ncRNA
AKT3	9	protein-coding	hsa-miR-619-5p	AKT3	-36.84	180	586	607	hsa-miR-619-5p	LOC102724927	-40.19	181	1757	1778	LOC102724927	1	ncRNA
AKT3	9	protein-coding	hsa-miR-619-5p	AKT3	-36.84	180	586	607	hsa-miR-619-5p	RHOQP2	-44.58	196	14917	14938	RHOQP2	9	pseudo
AKT3	9	protein-coding	hsa-miR-619-5p	AKT3	-36.84	180	586	607	hsa-miR-619-5p	RHOQP2	-42.14	192	10800	10821	RHOQP2	9	pseudo
AKT3	9	protein-coding	hsa-miR-619-5p	AKT3	-36.84	180	586	607	hsa-miR-619-5p	RHOQP2	-42.14	192	11167	11188	RHOQP2	9	pseudo
AKT3	9	protein-coding	hsa-miR-619-5p	AKT3	-36.84	180	586	607	hsa-miR-619-5p	RHOQP2	-37.61	184	15051	15072	RHOQP2	9	pseudo
AKT3	9	protein-coding	hsa-miR-619-5p	AKT3	-36.84	180	586	607	hsa-miR-619-5p	RHOQP2	-36.83	168	11299	11320	RHOQP2	9	pseudo
AKT3	9	protein-coding	hsa-miR-619-5p	AKT3	-36.84	180	586	607	hsa-miR-619-5p	ERLEC1P1	-47.05	200	23428	23449	ERLEC1P1	1	pseudo
AKT3	9	protein-coding	hsa-miR-619-5p	AKT3	-36.84	180	586	607	hsa-miR-619-5p	ERLEC1P1	-44.11	196	26915	26936	ERLEC1P1	1	pseudo
AKT3	9	protein-coding	hsa-miR-619-5p	AKT3	-36.84	180	586	607	hsa-miR-619-5p	ERLEC1P1	-42.14	192	14810	14831	ERLEC1P1	1	pseudo
AKT3	9	protein-coding	hsa-miR-619-5p	AKT3	-36.84	180	586	607	hsa-miR-619-5p	ERLEC1P1	-42.24	192	23546	23567	ERLEC1P1	1	pseudo
AKT3	9	protein-coding	hsa-miR-619-5p	AKT3	-36.84	180	586	607	hsa-miR-619-5p	ERLEC1P1	-38.9	192	45888	45909	ERLEC1P1	1	pseudo
AKT3	9	protein-coding	hsa-miR-619-5p	AKT3	-36.84	180	586	607	hsa-miR-619-5p	ERLEC1P1	-37.86	181	6437	6458	ERLEC1P1	1	pseudo
AKT3	9	protein-coding	hsa-miR-619-5p	AKT3	-36.84	180	586	607	hsa-miR-619-5p	ERLEC1P1	-35.12	177	8956	8977	ERLEC1P1	1	pseudo
AKT3	9	protein-coding	hsa-miR-619-5p	AKT3	-36.84	180	586	607	hsa-miR-619-5p	ERLEC1P1	-39.07	176	2394	2415	ERLEC1P1	1	pseudo
AKT3	9	protein-coding	hsa-miR-619-5p	AKT3	-36.84	180	586	607	hsa-miR-619-5p	ERLEC1P1	-34.58	176	24623	24644	ERLEC1P1	1	pseudo
AKT3	9	protein-coding	hsa-miR-619-5p	AKT3	-36.84	180	586	607	hsa-miR-619-5p	ERLEC1P1	-33.81	175	27062	27083	ERLEC1P1	1	pseudo
AKT3	9	protein-coding	hsa-miR-619-5p	AKT3	-36.84	180	586	607	hsa-miR-619-5p	ERLEC1P1	-35.87	172	8821	8842	ERLEC1P1	1	pseudo
AKT3	9	protein-coding	hsa-miR-619-5p	AKT3	-36.84	180	586	607	hsa-miR-619-5p	ERLEC1P1	-42.46	172	14946	14967	ERLEC1P1	1	pseudo
AKT3	9	protein-coding	hsa-miR-619-5p	AKT3	-36.84	180	586	607	hsa-miR-619-5p	ERLEC1P1	-29.8	172	15472	15493	ERLEC1P1	1	pseudo
AKT3	9	protein-coding	hsa-miR-619-5p	AKT3	-36.84	180	586	607	hsa-miR-619-5p	ERLEC1P1	-33.35	170	24757	24778	ERLEC1P1	1	pseudo
AKT3	9	protein-coding	hsa-miR-619-5p	AKT3	-36.84	180	586	607	hsa-miR-619-5p	ERLEC1P1	-39.72	170	55124	55145	ERLEC1P1	1	pseudo

AKT3	9	protein-coding	hsa-miR-619-5p	AKT3	-36.84	180	586	607	hsa-miR-619-5p	ERLEC1P1	-36.18	168	6303	6324	ERLEC1P1	1	pseudo
AKT3	9	protein-coding	hsa-miR-619-5p	AKT3	-36.84	180	586	607	hsa-miR-619-5p	ERLEC1P1	-32.38	164	34946	34967	ERLEC1P1	1	pseudo
AKT3	9	protein-coding	hsa-miR-619-5p	AKT3	-36.84	180	586	607	hsa-miR-619-5p	ERLEC1P1	-33.03	161	35069	35092	ERLEC1P1	1	pseudo
AKT3	9	protein-coding	hsa-miR-619-5p	AKT3	-36.84	180	586	607	hsa-miR-619-5p	ERLEC1P1	-30.53	160	21313	21334	ERLEC1P1	1	pseudo
AKT3	9	protein-coding	hsa-miR-619-5p	AKT3	-36.84	180	586	607	hsa-miR-619-5p	ZFP91-CNTF	-26.81	167	2270	2293	ZFP91-CNTF	9	ncRNA
AKT3	9	protein-coding	hsa-miR-619-5p	AKT3	-36.84	180	586	607	hsa-miR-619-5p	UBE2Q2P12	-41.77	188	6170	6191	UBE2Q2P12	1	pseudo
AKT3	9	protein-coding	hsa-miR-619-5p	AKT3	-36.84	180	586	607	hsa-miR-619-5p	UBE2Q2P11	-41.76	192	6163	6184	UBE2Q2P11	1	pseudo
AKT3	9	protein-coding	hsa-miR-619-5p	AKT3	-36.84	180	586	607	hsa-miR-619-5p	UBE2Q2P11	-40.78	180	6029	6050	UBE2Q2P11	1	pseudo
AKT3	9	protein-coding	hsa-miR-619-5p	AKT3	-36.84	180	586	607	hsa-miR-619-5p	LOC100130872	-37.51	184	4844	4865	LOC100130872	1	ncRNA
AKT3	9	protein-coding	hsa-miR-619-5p	AKT3	-36.84	180	586	607	hsa-miR-619-5p	SEPT7P3	-42.14	192	8390	8411	SEPT7P3	1	pseudo
AKT3	9	protein-coding	hsa-miR-619-5p	AKT3	-36.84	180	586	607	hsa-miR-619-5p	SEPT7P3	-38.34	174	6570	6591	SEPT7P3	1	pseudo
AKT3	9	protein-coding	hsa-miR-619-5p	AKT3	-36.84	180	586	607	hsa-miR-619-5p	SEPT7P3	-38.01	160	6435	6456	SEPT7P3	1	pseudo
AKT3	9	protein-coding	hsa-miR-619-5p	AKT3	-36.84	180	586	607	hsa-miR-619-5p	LOC100420851	-47.05	200	39183	39204	LOC100420851	1	pseudo
AKT3	9	protein-coding	hsa-miR-619-5p	AKT3	-36.84	180	586	607	hsa-miR-619-5p	LOC100420851	-44.84	200	62075	62096	LOC100420851	1	pseudo
AKT3	9	protein-coding	hsa-miR-619-5p	AKT3	-36.84	180	586	607	hsa-miR-619-5p	LOC100420851	-44.58	196	24308	24329	LOC100420851	1	pseudo
AKT3	9	protein-coding	hsa-miR-619-5p	AKT3	-36.84	180	586	607	hsa-miR-619-5p	LOC100420851	-42.14	192	29740	29761	LOC100420851	1	pseudo
AKT3	9	protein-coding	hsa-miR-619-5p	AKT3	-36.84	180	586	607	hsa-miR-619-5p	LOC100420851	-41.76	192	35608	35629	LOC100420851	1	pseudo
AKT3	9	protein-coding	hsa-miR-619-5p	AKT3	-36.84	180	586	607	hsa-miR-619-5p	LOC100420851	-42.14	192	64936	64957	LOC100420851	1	pseudo
AKT3	9	protein-coding	hsa-miR-619-5p	AKT3	-36.84	180	586	607	hsa-miR-619-5p	LOC100420851	-37.33	188	61236	61257	LOC100420851	1	pseudo
AKT3	9	protein-coding	hsa-miR-619-5p	AKT3	-36.84	180	586	607	hsa-miR-619-5p	LOC100420851	-38.15	186	64450	64471	LOC100420851	1	pseudo
AKT3	9	protein-coding	hsa-miR-619-5p	AKT3	-36.84	180	586	607	hsa-miR-619-5p	LOC100420851	-38.65	184	30421	30442	LOC100420851	1	pseudo
AKT3	9	protein-coding	hsa-miR-619-5p	AKT3	-36.84	180	586	607	hsa-miR-619-5p	LOC100420851	-38.65	184	54892	54913	LOC100420851	1	pseudo
AKT3	9	protein-coding	hsa-miR-619-5p	AKT3	-36.84	180	586	607	hsa-miR-619-5p	LOC100420851	-33.94	182	65070	65091	LOC100420851	1	pseudo
AKT3	9	protein-coding	hsa-miR-619-5p	AKT3	-36.84	180	586	607	hsa-miR-619-5p	LOC100420851	-30.94	178	61771	61792	LOC100420851	1	pseudo
AKT3	9	protein-coding	hsa-miR-619-5p	AKT3	-36.84	180	586	607	hsa-miR-619-5p	LOC100420851	-32.82	177	24441	24464	LOC100420851	1	pseudo
AKT3	9	protein-coding	hsa-miR-619-5p	AKT3	-36.84	180	586	607	hsa-miR-619-5p	LOC100420851	-41.97	176	39319	39340	LOC100420851	1	pseudo
AKT3	9	protein-coding	hsa-miR-619-5p	AKT3	-36.84	180	586	607	hsa-miR-619-5p	LOC100420851	-39.65	176	63386	63407	LOC100420851	1	pseudo
AKT3	9	protein-coding	hsa-miR-619-5p	AKT3	-36.84	180	586	607	hsa-miR-619-5p	LOC100420851	-39.03	172	55026	55047	LOC100420851	1	pseudo
AKT3	9	protein-coding	hsa-miR-619-5p	AKT3	-36.84	180	586	607	hsa-miR-619-5p	LOC100420851	-31.97	168	63251	63272	LOC100420851	1	pseudo
AKT3	9	protein-coding	hsa-miR-619-5p	AKT3	-36.84	180	586	607	hsa-miR-619-5p	LOC100420851	-29.52	164	35475	35496	LOC100420851	1	pseudo
AKT3	9	protein-coding	hsa-miR-619-5p	AKT3	-36.84	180	586	607	hsa-miR-619-5p	LOC100420851	-34.71	164	62211	62232	LOC100420851	1	pseudo
AKT3	9	protein-coding	hsa-miR-619-5p	AKT3	-36.84	180	586	607	hsa-miR-619-5p	LOC101926967	-38.65	184	1128	1149	LOC101926967	0	ncRNA
AKT3	9	protein-coding	hsa-miR-619-5p	AKT3	-36.84	180	586	607	hsa-miR-619-5p	KCNQ1OT1	-39.99	192	74144	74165	KCNQ1OT1	9	ncRNA
AKT3	9	protein-coding	hsa-miR-619-5p	AKT3	-36.84	180	586	607	hsa-miR-619-5p	KCNQ1OT1	-36.73	184	19821	19842	KCNQ1OT1	9	ncRNA
AKT3	9	protein-coding	hsa-miR-619-5p	AKT3	-36.84	180	586	607	hsa-miR-619-5p	KCNQ1OT1	-33.57	184	71961	71982	KCNQ1OT1	9	ncRNA

AKT3	9	protein-coding	hsa-miR-619-5p	AKT3	-36.84	180	586	607	hsa-miR-619-5p	KCNQ1OT1	-35.9	184	79631	79652	KCNQ1OT1	9	ncRNA
AKT3	9	protein-coding	hsa-miR-619-5p	AKT3	-36.84	180	586	607	hsa-miR-619-5p	KCNQ1OT1	-38.65	184	88501	88522	KCNQ1OT1	9	ncRNA
AKT3	9	protein-coding	hsa-miR-619-5p	AKT3	-36.84	180	586	607	hsa-miR-619-5p	KCNQ1OT1	-38.65	184	91224	91245	KCNQ1OT1	9	ncRNA
AKT3	9	protein-coding	hsa-miR-619-5p	AKT3	-36.84	180	586	607	hsa-miR-619-5p	KCNQ1OT1	-32.91	177	79499	79518	KCNQ1OT1	9	ncRNA
AKT3	9	protein-coding	hsa-miR-619-5p	AKT3	-36.84	180	586	607	hsa-miR-619-5p	KCNQ1OT1	-40.2	174	59562	59583	KCNQ1OT1	9	ncRNA
AKT3	9	protein-coding	hsa-miR-619-5p	AKT3	-36.84	180	586	607	hsa-miR-619-5p	KCNQ1OT1	-31.22	172	78872	78891	KCNQ1OT1	9	ncRNA
AKT3	9	protein-coding	hsa-miR-619-5p	AKT3	-36.84	180	586	607	hsa-miR-619-5p	KCNQ1OT1	-39.34	169	88628	88649	KCNQ1OT1	9	ncRNA
AKT3	9	protein-coding	hsa-miR-619-5p	AKT3	-36.84	180	586	607	hsa-miR-619-5p	KCNQ1OT1	-44.55	168	78735	78756	KCNQ1OT1	9	ncRNA
AKT3	9	protein-coding	hsa-miR-619-5p	AKT3	-36.84	180	586	607	hsa-miR-619-5p	KCNQ1OT1	-36.58	165	19954	19975	KCNQ1OT1	9	ncRNA
AKT3	9	protein-coding	hsa-miR-619-5p	AKT3	-36.84	180	586	607	hsa-miR-619-5p	KCNQ1OT1	-26.01	164	35536	35555	KCNQ1OT1	9	ncRNA
AKT3	9	protein-coding	hsa-miR-619-5p	AKT3	-36.84	180	586	607	hsa-miR-619-5p	KCNQ1OT1	-38.55	164	53197	53218	KCNQ1OT1	9	ncRNA
AKT3	9	protein-coding	hsa-miR-619-5p	AKT3	-36.84	180	586	607	hsa-miR-619-5p	KCNQ1OT1	-33.23	162	72097	72118	KCNQ1OT1	9	ncRNA
AKT3	9	protein-coding	hsa-miR-619-5p	AKT3	-36.84	180	586	607	hsa-miR-619-5p	KCNQ1OT1	-26.32	161	54794	54815	KCNQ1OT1	9	ncRNA
AKT3	9	protein-coding	hsa-miR-619-5p	AKT3	-36.84	180	586	607	hsa-miR-619-5p	HERC2P5	-47.05	200	9808	9829	HERC2P5	1	pseudo
AKT3	9	protein-coding	hsa-miR-619-5p	AKT3	-36.84	180	586	607	hsa-miR-619-5p	HERC2P5	-47.05	200	14187	14208	HERC2P5	1	pseudo
AKT3	9	protein-coding	hsa-miR-619-5p	AKT3	-36.84	180	586	607	hsa-miR-619-5p	HERC2P5	-47.05	200	32872	32893	HERC2P5	1	pseudo
AKT3	9	protein-coding	hsa-miR-619-5p	AKT3	-36.84	180	586	607	hsa-miR-619-5p	HERC2P5	-44.89	196	13891	13912	HERC2P5	1	pseudo
AKT3	9	protein-coding	hsa-miR-619-5p	AKT3	-36.84	180	586	607	hsa-miR-619-5p	HERC2P5	-42.14	192	455	476	HERC2P5	1	pseudo
AKT3	9	protein-coding	hsa-miR-619-5p	AKT3	-36.84	180	586	607	hsa-miR-619-5p	HERC2P5	-41.76	192	9947	9968	HERC2P5	1	pseudo
AKT3	9	protein-coding	hsa-miR-619-5p	AKT3	-36.84	180	586	607	hsa-miR-619-5p	HERC2P5	-37.86	181	33008	33029	HERC2P5	1	pseudo
AKT3	9	protein-coding	hsa-miR-619-5p	AKT3	-36.84	180	586	607	hsa-miR-619-5p	HERC2P5	-37.41	162	24900	24921	HERC2P5	1	pseudo
AKT3	9	protein-coding	hsa-miR-619-5p	AKT3	-36.84	180	586	607	hsa-miR-619-5p	HERC2P5	-30.01	161	14322	14343	HERC2P5	1	pseudo
AKT3	9	protein-coding	hsa-miR-619-5p	AKT3	-36.84	180	586	607	hsa-miR-619-5p	CSPG4P12	-41.42	184	7592	7613	CSPG4P12	1	pseudo
AKT3	9	protein-coding	hsa-miR-619-5p	AKT3	-36.84	180	586	607	hsa-miR-619-5p	CSPG4P12	-33.4	168	841	862	CSPG4P12	1	pseudo
AKT3	9	protein-coding	hsa-miR-619-5p	AKT3	-36.84	180	586	607	hsa-miR-619-5p	CSPG4P12	-35	164	6618	6639	CSPG4P12	1	pseudo
AKT3	9	protein-coding	hsa-miR-619-5p	AKT3	-36.84	180	586	607	hsa-miR-619-5p	CSPG4P10	-41.42	184	7566	7587	CSPG4P10	1	pseudo
AKT3	9	protein-coding	hsa-miR-619-5p	AKT3	-36.84	180	586	607	hsa-miR-619-5p	CSPG4P10	-33.4	168	815	836	CSPG4P10	1	pseudo
AKT3	9	protein-coding	hsa-miR-619-5p	AKT3	-36.84	180	586	607	hsa-miR-619-5p	CSPG4P10	-35	164	6592	6613	CSPG4P10	1	pseudo
AKT3	9	protein-coding	hsa-miR-619-5p	AKT3	-36.84	180	586	607	hsa-miR-619-5p	CSPG4P11	-33.4	168	884	905	CSPG4P11	1	pseudo
AKT3	9	protein-coding	hsa-miR-619-5p	AKT3	-36.84	180	586	607	hsa-miR-619-5p	ASAP1-IT2	-42.14	192	690	711	ASAP1-IT2	9	ncRNA
AKT3	9	protein-coding	hsa-miR-619-5p	AKT3	-36.84	180	586	607	hsa-miR-619-5p	ASAP1-IT2	-38.65	184	1925	1946	ASAP1-IT2	9	ncRNA
AKT3	9	protein-coding	hsa-miR-619-5p	AKT3	-36.84	180	586	607	hsa-miR-619-5p	ASAP1-IT2	-39.53	176	825	846	ASAP1-IT2	9	ncRNA
AKT3	9	protein-coding	hsa-miR-619-5p	AKT3	-36.84	180	586	607	hsa-miR-619-5p	PRKY	-39.61	192	5176	5197	PRKY	1	pseudo
AKT3	9	protein-coding	hsa-miR-619-5p	AKT3	-36.84	180	586	607	hsa-miR-619-5p	PRKY	-34.38	187	5040	5061	PRKY	1	pseudo
AKT3	9	protein-coding	hsa-miR-619-5p	AKT3	-36.84	180	586	607	hsa-miR-619-5p	PRKY	-35.93	168	2107	2128	PRKY	1	pseudo

AKT3	9	protein-coding	hsa-miR-619-5p	AKT3	-36.84	180	586	607	hsa-miR-619-5p	PRKY	-32.29	166	2251	2272	PRKY	1	pseudo
AKT3	9	protein-coding	hsa-miR-619-5p	AKT3	-36.84	180	586	607	hsa-miR-619-5p	SLC35E1P1	-35.93	180	2410	2431	SLC35E1P1	9	pseudo
AKT3	9	protein-coding	hsa-miR-619-5p	AKT3	-36.84	180	586	607	hsa-miR-619-5p	SLC35E1P1	-39.26	160	2532	2553	SLC35E1P1	9	pseudo
AKT3	9	protein-coding	hsa-miR-619-5p	AKT3	-36.84	180	586	607	hsa-miR-619-5p	LOC100419773	-47.05	200	1301	1322	LOC100419773	9	pseudo
AKT3	9	protein-coding	hsa-miR-619-5p	AKT3	-36.84	180	586	607	hsa-miR-619-5p	ZNF702P	-38.65	184	2459	2480	ZNF702P	1	pseudo
AKT3	9	protein-coding	hsa-miR-619-5p	AKT3	-36.84	180	586	607	hsa-miR-619-5p	LOC101928139	-32.38	160	1799	1820	LOC101928139	1	ncRNA
AKT3	9	protein-coding	hsa-miR-619-5p	AKT3	-36.84	180	586	607	hsa-miR-619-5p	RPL21P44	-38.73	184	1470	1491	RPL21P44	9	pseudo
AKT3	9	protein-coding	hsa-miR-619-5p	AKT3	-36.84	180	586	607	hsa-miR-619-5p	RPL21P44	-29.05	167	1676	1697	RPL21P44	9	pseudo
AKT3	9	protein-coding	hsa-miR-619-5p	AKT3	-36.84	180	586	607	hsa-miR-619-5p	LINC01000	-42.14	192	4791	4812	LINC01000	1	ncRNA
AKT3	9	protein-coding	hsa-miR-619-5p	AKT3	-36.84	180	586	607	hsa-miR-619-5p	LINC01000	-34.66	184	8724	8745	LINC01000	1	ncRNA
AKT3	9	protein-coding	hsa-miR-619-5p	AKT3	-36.84	180	586	607	hsa-miR-619-5p	FLJ32255	-35.59	175	263	284	FLJ32255	0	ncRNA
AKT3	9	protein-coding	hsa-miR-619-5p	AKT3	-36.84	180	586	607	hsa-miR-619-5p	LOC653406	-38.65	184	3877	3898	LOC653406	1	pseudo
AKT3	9	protein-coding	hsa-miR-619-5p	AKT3	-36.84	180	586	607	hsa-miR-619-5p	LOC653406	-41.97	176	4012	4033	LOC653406	1	pseudo
AKT3	9	protein-coding	hsa-miR-619-5p	AKT3	-36.84	180	586	607	hsa-miR-619-5p	GLULP2	-39.49	192	1271	1292	GLULP2	9	pseudo
AKT3	9	protein-coding	hsa-miR-619-5p	AKT3	-36.84	180	586	607	hsa-miR-619-5p	GLULP2	-44.22	184	2170	2191	GLULP2	9	pseudo
AKT3	9	protein-coding	hsa-miR-619-5p	AKT3	-36.84	180	586	607	hsa-miR-619-5p	MBNL1-AS1	-38.65	184	2061	2082	MBNL1-AS1	1	ncRNA
AKT3	9	protein-coding	hsa-miR-619-5p	AKT3	-36.84	180	586	607	hsa-miR-619-5p	LOC440300	-42.14	192	7480	7501	LOC440300	1	pseudo
AKT3	9	protein-coding	hsa-miR-619-5p	AKT3	-36.84	180	586	607	hsa-miR-619-5p	LOC440300	-38.65	184	4326	4347	LOC440300	1	pseudo
AKT3	9	protein-coding	hsa-miR-619-5p	AKT3	-36.84	180	586	607	hsa-miR-619-5p	LOC440300	-38.35	181	4461	4482	LOC440300	1	pseudo
AKT3	9	protein-coding	hsa-miR-619-5p	AKT3	-36.84	180	586	607	hsa-miR-619-5p	ZNF37BP	-32.11	176	4100	4121	ZNF37BP	9	pseudo
AKT3	9	protein-coding	hsa-miR-619-5p	AKT3	-36.84	180	586	607	hsa-miR-619-5p	ZNF37BP	-36.62	160	5915	5936	ZNF37BP	9	pseudo
AKT3	9	protein-coding	hsa-miR-619-5p	AKT3	-36.84	180	586	607	hsa-miR-619-5p	KLF3P1	-33.98	177	1490	1511	KLF3P1	0	pseudo
AKT3	9	protein-coding	hsa-miR-619-5p	AKT3	-36.84	180	586	607	hsa-miR-619-5p	KLF3P1	-35.87	172	1358	1379	KLF3P1	0	pseudo
AKT3	9	protein-coding	hsa-miR-619-5p	AKT3	-36.84	180	586	607	hsa-miR-619-5p	MIRLET7BHG	-47.05	200	2160	2181	MIRLET7BHG	1	ncRNA
AKT3	9	protein-coding	hsa-miR-619-5p	AKT3	-36.84	180	586	607	hsa-miR-619-5p	WAC-AS1	-39.99	190	4020	4041	WAC-AS1	9	ncRNA
AKT3	9	protein-coding	hsa-miR-619-5p	AKT3	-36.84	180	586	607	hsa-miR-619-5p	WAC-AS1	-39.9	182	2161	2182	WAC-AS1	9	ncRNA
AKT3	9	protein-coding	hsa-miR-619-5p	AKT3	-36.84	180	586	607	hsa-miR-619-5p	WAC-AS1	-40.78	180	2026	2047	WAC-AS1	9	ncRNA
AKT3	9	protein-coding	hsa-miR-619-5p	AKT3	-36.84	180	586	607	hsa-miR-619-5p	WAC-AS1	-42.35	176	3886	3907	WAC-AS1	9	ncRNA
AKT3	9	protein-coding	hsa-miR-619-5p	AKT3	-36.84	180	586	607	hsa-miR-619-5p	WAC-AS1	-41.5	168	1560	1581	WAC-AS1	9	ncRNA
AKT3	9	protein-coding	hsa-miR-619-5p	AKT3	-36.84	180	586	607	hsa-miR-619-5p	DNM1P46	-39.9	184	3563	3584	DNM1P46	1	pseudo
AKT3	9	protein-coding	hsa-miR-619-5p	AKT3	-36.84	180	586	607	hsa-miR-619-5p	DNM1P46	-35.79	181	3698	3719	DNM1P46	1	pseudo
AKT3	9	protein-coding	hsa-miR-619-5p	AKT3	-36.84	180	586	607	hsa-miR-619-5p	UBE2Q2P6	-41.76	192	6145	6166	UBE2Q2P6	1	pseudo
AKT3	9	protein-coding	hsa-miR-619-5p	AKT3	-36.84	180	586	607	hsa-miR-619-5p	UBE2Q2P6	-40.78	180	6011	6032	UBE2Q2P6	1	pseudo
AKT3	9	protein-coding	hsa-miR-619-5p	AKT3	-36.84	180	586	607	hsa-miR-619-5p	ZNF252P	-34.16	184	3919	3940	ZNF252P	9	pseudo
AKT3	9	protein-coding	hsa-miR-619-5p	AKT3	-36.84	180	586	607	hsa-miR-619-5p	ZNF252P	-35.29	166	4050	4071	ZNF252P	9	pseudo

AKT3	9	protein-coding	hsa-miR-619-5p	AKT3	-36.84	180	586	607	hsa-miR-619-5p	LOC100287825	-47.05	200	7978	7999	LOC100287825	9	pseudo
AKT3	9	protein-coding	hsa-miR-619-5p	AKT3	-36.84	180	586	607	hsa-miR-619-5p	LOC100287825	-34.14	165	8113	8134	LOC100287825	9	pseudo
AKT3	9	protein-coding	hsa-miR-619-5p	AKT3	-36.84	180	586	607	hsa-miR-619-5p	LOC100420571	-47.05	200	492	513	LOC100420571	1	pseudo
AKT3	9	protein-coding	hsa-miR-619-5p	AKT3	-36.84	180	586	607	hsa-miR-619-5p	LOC100420571	-35.12	175	627	648	LOC100420571	1	pseudo
AKT3	9	protein-coding	hsa-miR-619-5p	AKT3	-36.84	180	586	607	hsa-miR-619-5p	LINC00476	-37.84	190	660	681	LINC00476	0	ncRNA
AKT3	9	protein-coding	hsa-miR-619-5p	AKT3	-36.84	180	586	607	hsa-miR-619-5p	LINC00476	-40.9	184	526	547	LINC00476	0	ncRNA
AKT3	9	protein-coding	hsa-miR-619-5p	AKT3	-36.84	180	586	607	hsa-miR-619-5p	LOC728519	-38.65	184	4540	4561	LOC728519	1	pseudo
AKT3	9	protein-coding	hsa-miR-619-5p	AKT3	-36.84	180	586	607	hsa-miR-619-5p	LOC728519	-41.97	176	4675	4696	LOC728519	1	pseudo
AKT3	9	protein-coding	hsa-miR-619-5p	AKT3	-36.84	180	586	607	hsa-miR-619-5p	PSMD5-AS1	-36.49	180	2964	2985	PSMD5-AS1	1	ncRNA
AKT3	9	protein-coding	hsa-miR-619-5p	AKT3	-36.84	180	586	607	hsa-miR-619-5p	PSMD5-AS1	-36.11	168	3097	3118	PSMD5-AS1	1	ncRNA
AKT3	9	protein-coding	hsa-miR-619-5p	AKT3	-36.84	180	586	607	hsa-miR-619-5p	LINC00327	-47.05	200	1674	1695	LINC00327	1	ncRNA
AKT3	9	protein-coding	hsa-miR-619-5p	AKT3	-36.84	180	586	607	hsa-miR-619-5p	C1ORF220	-34.66	184	1192	1213	C1ORF220	1	ncRNA
AKT3	9	protein-coding	hsa-miR-619-5p	AKT3	-36.84	180	586	607	hsa-miR-619-5p	C1ORF220	-35.3	174	1329	1350	C1ORF220	1	ncRNA
AKT3	9	protein-coding	hsa-miR-619-5p	AKT3	-36.84	180	586	607	hsa-miR-619-5p	FRMD6-AS1	-42.14	192	1064	1085	FRMD6-AS1	9	ncRNA
AKT3	9	protein-coding	hsa-miR-619-5p	AKT3	-36.84	180	586	607	hsa-miR-619-5p	ZNF271	-35.87	172	2342	2363	ZNF271	9	pseudo
AKT3	9	protein-coding	hsa-miR-4656	AKT3	-35.06	172	32	53	hsa-miR-4656	KIAA1656	-34.27	161	5593	5616	KIAA1656	1	ncRNA
AKT3	9	protein-coding	hsa-miR-4656	AKT3	-35.06	172	32	53	hsa-miR-4656	LOC390933	-37.55	182	334	356	LOC390933	0	pseudo
AKT3	9	protein-coding	hsa-miR-4656	AKT3	-35.06	172	32	53	hsa-miR-4656	FAM13A-AS1	-35.99	172	876	899	FAM13A-AS1	1	ncRNA
AKT3	9	protein-coding	hsa-miR-4656	AKT3	-35.06	172	32	53	hsa-miR-4656	LOC102724814	-33.04	160	163	186	LOC102724814	0	ncRNA
AKT3	9	protein-coding	hsa-miR-4656	AKT3	-35.06	172	32	53	hsa-miR-4656	LOC102724814	-33.58	160	887	908	LOC102724814	0	ncRNA
AKT3	9	protein-coding	hsa-miR-4656	AKT3	-35.06	172	32	53	hsa-miR-4656	CD27-AS1	-33.94	166	662	685	CD27-AS1	0	ncRNA
AKT3	9	protein-coding	hsa-miR-4656	AKT3	-35.06	172	32	53	hsa-miR-4656	LINC00674	-28.19	167	10780	10802	LINC00674	9	ncRNA
AKT3	9	protein-coding	hsa-miR-4656	AKT3	-35.06	172	32	53	hsa-miR-4656	IL6STP1	-33.63	165	3604	3625	IL6STP1	1	pseudo
AKT3	9	protein-coding	hsa-miR-4656	AKT3	-35.06	172	32	53	hsa-miR-4656	IL6STP1	-33.33	163	4570	4591	IL6STP1	1	pseudo
AKT3	9	protein-coding	hsa-miR-4656	AKT3	-35.06	172	32	53	hsa-miR-4656	LOC101060398	-35.34	176	620	641	LOC101060398	9	ncRNA
AKT3	9	protein-coding	hsa-miR-4656	AKT3	-35.06	172	32	53	hsa-miR-4656	LINC00886	-35.34	176	1712	1733	LINC00886	1	ncRNA
AKT3	9	protein-coding	hsa-miR-4656	AKT3	-35.06	172	32	53	hsa-miR-4656	UBE2Q2P12	-34.35	160	4921	4942	UBE2Q2P12	1	pseudo
AKT3	9	protein-coding	hsa-miR-4656	AKT3	-35.06	172	32	53	hsa-miR-4656	UBE2Q2P11	-34.35	160	4917	4938	UBE2Q2P11	1	pseudo
AKT3	9	protein-coding	hsa-miR-4656	AKT3	-35.06	172	32	53	hsa-miR-4656	LINC00654	-32.34	167	320	343	LINC00654	1	ncRNA
AKT3	9	protein-coding	hsa-miR-4656	AKT3	-35.06	172	32	53	hsa-miR-4656	LOC100420851	-29.36	167	15287	15309	LOC100420851	1	pseudo
AKT3	9	protein-coding	hsa-miR-4656	AKT3	-35.06	172	32	53	hsa-miR-4656	LOC100420851	-34.92	160	3755	3776	LOC100420851	1	pseudo
AKT3	9	protein-coding	hsa-miR-4656	AKT3	-35.06	172	32	53	hsa-miR-4656	KCNQ1OT1	-31.7	165	9905	9929	KCNQ1OT1	9	ncRNA
AKT3	9	protein-coding	hsa-miR-4656	AKT3	-35.06	172	32	53	hsa-miR-4656	KCNQ1OT1	-33.45	164	35653	35677	KCNQ1OT1	9	ncRNA
AKT3	9	protein-coding	hsa-miR-4656	AKT3	-35.06	172	32	53	hsa-miR-4656	KCNQ1OT1	-27.54	163	31842	31864	KCNQ1OT1	9	ncRNA
AKT3	9	protein-coding	hsa-miR-4656	AKT3	-35.06	172	32	53	hsa-miR-4656	PAXIP1-AS2	-33.17	160	616	638	PAXIP1-AS2	0	ncRNA

AKT3	9	protein-coding	hsa-miR-4656	AKT3	-35.06	172	32	53	hsa-miR-4656	FUT8-AS1	-28.88	165	325	349	FUT8-AS1	0	ncRNA
AKT3	9	protein-coding	hsa-miR-4656	AKT3	-35.06	172	32	53	hsa-miR-4656	TAPT1-AS1	-28.91	160	1972	1995	TAPT1-AS1	1	ncRNA
AKT3	9	protein-coding	hsa-miR-4656	AKT3	-35.06	172	32	53	hsa-miR-4656	LINC01000	-32.47	172	8461	8482	LINC01000	1	ncRNA
AKT3	9	protein-coding	hsa-miR-4656	AKT3	-35.06	172	32	53	hsa-miR-4656	FLJ32255	-34.68	168	1750	1775	FLJ32255	0	ncRNA
AKT3	9	protein-coding	hsa-miR-4656	AKT3	-35.06	172	32	53	hsa-miR-4656	ZSCAN16-AS1	-27.7	162	1459	1481	ZSCAN16-AS1	1	ncRNA
AKT3	9	protein-coding	hsa-miR-4656	AKT3	-35.06	172	32	53	hsa-miR-4656	LOC148430	-27.49	164	691	714	LOC148430	1	pseudo
AKT3	9	protein-coding	hsa-miR-4656	AKT3	-35.06	172	32	53	hsa-miR-4656	LINC00954	-32.97	170	1459	1479	LINC00954	1	ncRNA
AKT3	9	protein-coding	hsa-miR-4656	AKT3	-35.06	172	32	53	hsa-miR-4656	HYMAI	-28.55	162	398	421	HYMAI	1	ncRNA
AKT3	9	protein-coding	hsa-miR-4656	AKT3	-35.06	172	32	53	hsa-miR-4656	MIRLET7BHG	-33	166	3440	3462	MIRLET7BHG	1	ncRNA
AKT3	9	protein-coding	hsa-miR-4656	AKT3	-35.06	172	32	53	hsa-miR-4656	MIRLET7BHG	-30.13	162	1482	1505	MIRLET7BHG	1	ncRNA
AKT3	9	protein-coding	hsa-miR-4656	AKT3	-35.06	172	32	53	hsa-miR-4656	WAC-AS1	-32.87	166	2966	2988	WAC-AS1	9	ncRNA
AKT3	9	protein-coding	hsa-miR-4656	AKT3	-35.06	172	32	53	hsa-miR-4656	WAC-AS1	-35.93	161	263	285	WAC-AS1	9	ncRNA
AKT3	9	protein-coding	hsa-miR-4656	AKT3	-35.06	172	32	53	hsa-miR-4656	MEIS3P1	-33.33	163	714	735	MEIS3P1	1	pseudo
AKT3	9	protein-coding	hsa-miR-4656	AKT3	-35.06	172	32	53	hsa-miR-4656	UBE2Q2P6	-34.35	160	4899	4920	UBE2Q2P6	1	pseudo
AKT3	9	protein-coding	hsa-miR-4656	AKT3	-35.06	172	32	53	hsa-miR-4656	LINC00565	-30.81	160	1704	1727	LINC00565	1	ncRNA
AKT3	9	protein-coding	hsa-miR-4656	AKT3	-35.06	172	32	53	hsa-miR-4656	LINC00327	-30.03	167	966	987	LINC00327	1	ncRNA
AKT3	9	protein-coding	hsa-miR-6780a-5p	AKT3	-26.51	164	167	190	hsa-miR-6780a-5p	LPP-AS2	-28.1	166	1527	1553	LPP-AS2	1	ncRNA
AKT3	9	protein-coding	hsa-miR-6780a-5p	AKT3	-26.51	164	167	190	hsa-miR-6780a-5p	LOC101928673	-25.06	162	1267	1287	LOC101928673	1	ncRNA
AKT3	9	protein-coding	hsa-miR-6780a-5p	AKT3	-26.51	164	167	190	hsa-miR-6780a-5p	ERLEC1P1	-28.83	172	18548	18571	ERLEC1P1	1	pseudo
AKT3	9	protein-coding	hsa-miR-6780a-5p	AKT3	-26.51	164	167	190	hsa-miR-6780a-5p	ERLEC1P1	-28.6	169	13189	13212	ERLEC1P1	1	pseudo
AKT3	9	protein-coding	hsa-miR-6780a-5p	AKT3	-26.51	164	167	190	hsa-miR-6780a-5p	ERLEC1P1	-27.86	168	10330	10353	ERLEC1P1	1	pseudo
AKT3	9	protein-coding	hsa-miR-6780a-5p	AKT3	-26.51	164	167	190	hsa-miR-6780a-5p	ERLEC1P1	-28.69	160	6682	6705	ERLEC1P1	1	pseudo
AKT3	9	protein-coding	hsa-miR-6780a-5p	AKT3	-26.51	164	167	190	hsa-miR-6780a-5p	ERLEC1P1	-28.23	160	8263	8286	ERLEC1P1	1	pseudo
AKT3	9	protein-coding	hsa-miR-6780a-5p	AKT3	-26.51	164	167	190	hsa-miR-6780a-5p	IPW	-28.22	163	3156	3180	IPW	1	ncRNA
AKT3	9	protein-coding	hsa-miR-6780a-5p	AKT3	-26.51	164	167	190	hsa-miR-6780a-5p	LINC00886	-26.51	164	1849	1872	LINC00886	1	ncRNA
AKT3	9	protein-coding	hsa-miR-6780a-5p	AKT3	-26.51	164	167	190	hsa-miR-6780a-5p	LOC100130872	-27.58	163	2802	2825	LOC100130872	1	ncRNA
AKT3	9	protein-coding	hsa-miR-6780a-5p	AKT3	-26.51	164	167	190	hsa-miR-6780a-5p	SEPT7P3	-28.67	170	9862	9884	SEPT7P3	1	pseudo
AKT3	9	protein-coding	hsa-miR-6780a-5p	AKT3	-26.51	164	167	190	hsa-miR-6780a-5p	LOC100420851	-27.54	172	23508	23531	LOC100420851	1	pseudo
AKT3	9	protein-coding	hsa-miR-6780a-5p	AKT3	-26.51	164	167	190	hsa-miR-6780a-5p	LOC100420851	-25.13	164	48318	48341	LOC100420851	1	pseudo
AKT3	9	protein-coding	hsa-miR-6780a-5p	AKT3	-26.51	164	167	190	hsa-miR-6780a-5p	LOC100420851	-25.64	163	25384	25405	LOC100420851	1	pseudo
AKT3	9	protein-coding	hsa-miR-6780a-5p	AKT3	-26.51	164	167	190	hsa-miR-6780a-5p	LOC100420851	-25.97	162	40914	40937	LOC100420851	1	pseudo
AKT3	9	protein-coding	hsa-miR-6780a-5p	AKT3	-26.51	164	167	190	hsa-miR-6780a-5p	KCNQ1OT1	-25.46	166	7271	7293	KCNQ1OT1	9	ncRNA
AKT3	9	protein-coding	hsa-miR-6780a-5p	AKT3	-26.51	164	167	190	hsa-miR-6780a-5p	KCNQ1OT1	-31.1	162	4359	4380	KCNQ1OT1	9	ncRNA
AKT3	9	protein-coding	hsa-miR-6780a-5p	AKT3	-26.51	164	167	190	hsa-miR-6780a-5p	KCNQ1OT1	-25.09	160	13696	13718	KCNQ1OT1	9	ncRNA
AKT3	9	protein-coding	hsa-miR-6780a-5p	AKT3	-26.51	164	167	190	hsa-miR-6780a-5p	HERC2P5	-27.6	164	24098	24121	HERC2P5	1	pseudo

AKT3	9	protein-coding	hsa-miR-6780a-5p	AKT3	-26.51	164	167	190	hsa-miR-6780a-5p	CSPG4P12	-31.6	176	9338	9361	CSPG4P12	1	pseudo
AKT3	9	protein-coding	hsa-miR-6780a-5p	AKT3	-26.51	164	167	190	hsa-miR-6780a-5p	CSPG4P10	-31.6	176	9312	9335	CSPG4P10	1	pseudo
AKT3	9	protein-coding	hsa-miR-6780a-5p	AKT3	-26.51	164	167	190	hsa-miR-6780a-5p	LINC00968	-26.68	160	887	909	LINC00968	1	ncRNA
AKT3	9	protein-coding	hsa-miR-6780a-5p	AKT3	-26.51	164	167	190	hsa-miR-6780a-5p	LINC01001	-28.22	167	1599	1622	LINC01001	9	ncRNA
AKT3	9	protein-coding	hsa-miR-6780a-5p	AKT3	-26.51	164	167	190	hsa-miR-6780a-5p	LINC01001	-28.22	167	4028	4051	LINC01001	9	ncRNA
AKT3	9	protein-coding	hsa-miR-6780a-5p	AKT3	-26.51	164	167	190	hsa-miR-6780a-5p	LINC01001	-28.49	163	1245	1268	LINC01001	9	ncRNA
AKT3	9	protein-coding	hsa-miR-6780a-5p	AKT3	-26.51	164	167	190	hsa-miR-6780a-5p	LINC01001	-28.44	161	412	432	LINC01001	9	ncRNA
AKT3	9	protein-coding	hsa-miR-6780a-5p	AKT3	-26.51	164	167	190	hsa-miR-6780a-5p	LINC01000	-28.22	167	1810	1833	LINC01000	1	ncRNA
AKT3	9	protein-coding	hsa-miR-6780a-5p	AKT3	-26.51	164	167	190	hsa-miR-6780a-5p	LINC01000	-29.36	167	3797	3820	LINC01000	1	ncRNA
AKT3	9	protein-coding	hsa-miR-6780a-5p	AKT3	-26.51	164	167	190	hsa-miR-6780a-5p	LINC01000	-28.49	163	1456	1479	LINC01000	1	ncRNA
AKT3	9	protein-coding	hsa-miR-6780a-5p	AKT3	-26.51	164	167	190	hsa-miR-6780a-5p	LINC01000	-28.44	161	638	658	LINC01000	1	ncRNA
AKT3	9	protein-coding	hsa-miR-6780a-5p	AKT3	-26.51	164	167	190	hsa-miR-6780a-5p	GLULP2	-30.59	161	3041	3063	GLULP2	9	pseudo
AKT3	9	protein-coding	hsa-miR-6780a-5p	AKT3	-26.51	164	167	190	hsa-miR-6780a-5p	GLULP2	-38.97	160	1187	1211	GLULP2	9	pseudo
AKT3	9	protein-coding	hsa-miR-6780a-5p	AKT3	-26.51	164	167	190	hsa-miR-6780a-5p	TMEM198B	-27.9	162	2592	2615	TMEM198B	1	pseudo
AKT3	9	protein-coding	hsa-miR-6780a-5p	AKT3	-26.51	164	167	190	hsa-miR-6780a-5p	LOC101928042	-32.81	176	5528	5551	LOC101928042	0	ncRNA
AKT3	9	protein-coding	hsa-miR-6780a-5p	AKT3	-26.51	164	167	190	hsa-miR-6780a-5p	USP32P2	-31.44	168	3464	3488	USP32P2	1	pseudo
AKT3	9	protein-coding	hsa-miR-6780a-5p	AKT3	-26.51	164	167	190	hsa-miR-6780a-5p	MIRLET7BHG	-30.9	160	3452	3474	MIRLET7BHG	1	ncRNA
AKT3	9	protein-coding	hsa-miR-6780a-5p	AKT3	-26.51	164	167	190	hsa-miR-6780a-5p	LINC00595	-32.1	167	613	636	LINC00595	1	ncRNA
AKT3	9	protein-coding	hsa-miR-6780a-5p	AKT3	-26.51	164	167	190	hsa-miR-6780a-5p	LOC101929719	-36.48	164	1677	1700	LOC101929719	1	ncRNA
AKT3	9	protein-coding	hsa-miR-6780a-5p	AKT3	-26.51	164	167	190	hsa-miR-6780a-5p	LOC100287825	-27.47	168	214	237	LOC100287825	9	pseudo
AKT3	9	protein-coding	hsa-miR-6780a-5p	AKT3	-26.51	164	167	190	hsa-miR-6780a-5p	HTR7P1	-28.69	160	3572	3595	HTR7P1	0	pseudo
AKT3	9	protein-coding	hsa-miR-6780a-5p	AKT3	-26.51	164	167	190	hsa-miR-6780a-5p	LOC100506730	-25.92	164	2104	2127	LOC100506730	9	ncRNA
AKT3	9	protein-coding	hsa-miR-6780a-5p	AKT3	-26.51	164	167	190	hsa-miR-6780a-5p	PSMD5-AS1	-26.79	165	843	867	PSMD5-AS1	1	ncRNA
AKT3	9	protein-coding	hsa-miR-6780a-5p	AKT3	-26.51	164	167	190	hsa-miR-6780a-5p	PSMD5-AS1	-30.16	160	3394	3418	PSMD5-AS1	1	ncRNA
AKT3	9	protein-coding	hsa-miR-6780a-5p	AKT3	-26.51	164	167	190	hsa-miR-6780a-5p	LINC00327	-27.92	165	775	798	LINC00327	1	ncRNA
AKT3	9	protein-coding	hsa-miR-6780a-5p	AKT3	-26.51	164	167	190	hsa-miR-6780a-5p	CROCCP3	-27.86	168	525	548	CROCCP3	0	pseudo
AKT3	9	protein-coding	hsa-miR-6780a-5p	AKT3	-26.51	164	167	190	hsa-miR-6780a-5p	RBMS1P1	-29.95	164	1929	1952	RBMS1P1	0	pseudo
AKT3	9	protein-coding	hsa-miR-7851-3p	AKT3	-32.29	160	7	28	hsa-miR-7851-3p	RHOQP2	-37.25	176	12054	12075	RHOQP2	9	pseudo
AKT3	9	protein-coding	hsa-miR-7851-3p	AKT3	-32.29	160	7	28	hsa-miR-7851-3p	RHOQP2	-32.31	168	14597	14618	RHOQP2	9	pseudo
AKT3	9	protein-coding	hsa-miR-7851-3p	AKT3	-32.29	160	7	28	hsa-miR-7851-3p	ERLEC1P1	-34.02	184	7081	7102	ERLEC1P1	1	pseudo
AKT3	9	protein-coding	hsa-miR-7851-3p	AKT3	-32.29	160	7	28	hsa-miR-7851-3p	ERLEC1P1	-34.02	184	46661	46682	ERLEC1P1	1	pseudo
AKT3	9	protein-coding	hsa-miR-7851-3p	AKT3	-32.29	160	7	28	hsa-miR-7851-3p	ERLEC1P1	-33.75	180	8236	8257	ERLEC1P1	1	pseudo
AKT3	9	protein-coding	hsa-miR-7851-3p	AKT3	-32.29	160	7	28	hsa-miR-7851-3p	ERLEC1P1	-32	176	10168	10189	ERLEC1P1	1	pseudo
AKT3	9	protein-coding	hsa-miR-7851-3p	AKT3	-32.29	160	7	28	hsa-miR-7851-3p	ERLEC1P1	-28.73	176	18386	18407	ERLEC1P1	1	pseudo
AKT3	9	protein-coding	hsa-miR-7851-3p	AKT3	-32.29	160	7	28	hsa-miR-7851-3p	ERLEC1P1	-28.83	176	21847	21868	ERLEC1P1	1	pseudo

AKT3	9	protein-coding	hsa-miR-7851-3p	AKT3	-32.29	160	7	28	hsa-miR-7851-3p	ERLEC1P1	-29.19	176	52054	52075	ERLEC1P1	1	pseudo
AKT3	9	protein-coding	hsa-miR-7851-3p	AKT3	-32.29	160	7	28	hsa-miR-7851-3p	ERLEC1P1	-25.44	167	45589	45610	ERLEC1P1	1	pseudo
AKT3	9	protein-coding	hsa-miR-7851-3p	AKT3	-32.29	160	7	28	hsa-miR-7851-3p	ERLEC1P1	-26.99	164	21797	21818	ERLEC1P1	1	pseudo
AKT3	9	protein-coding	hsa-miR-7851-3p	AKT3	-32.29	160	7	28	hsa-miR-7851-3p	ERLEC1P1	-31.36	160	6655	6676	ERLEC1P1	1	pseudo
AKT3	9	protein-coding	hsa-miR-7851-3p	AKT3	-32.29	160	7	28	hsa-miR-7851-3p	IL6STP1	-28.54	160	2613	2634	IL6STP1	1	pseudo
AKT3	9	protein-coding	hsa-miR-7851-3p	AKT3	-32.29	160	7	28	hsa-miR-7851-3p	UBE2Q2P12	-34.02	184	5244	5265	UBE2Q2P12	1	pseudo
AKT3	9	protein-coding	hsa-miR-7851-3p	AKT3	-32.29	160	7	28	hsa-miR-7851-3p	UBE2Q2P12	-26.13	167	1335	1356	UBE2Q2P12	1	pseudo
AKT3	9	protein-coding	hsa-miR-7851-3p	AKT3	-32.29	160	7	28	hsa-miR-7851-3p	UBE2Q2P12	-29.61	163	6595	6616	UBE2Q2P12	1	pseudo
AKT3	9	protein-coding	hsa-miR-7851-3p	AKT3	-32.29	160	7	28	hsa-miR-7851-3p	UBE2Q2P11	-34.02	184	5240	5261	UBE2Q2P11	1	pseudo
AKT3	9	protein-coding	hsa-miR-7851-3p	AKT3	-32.29	160	7	28	hsa-miR-7851-3p	UBE2Q2P11	-26.13	167	1321	1342	UBE2Q2P11	1	pseudo
AKT3	9	protein-coding	hsa-miR-7851-3p	AKT3	-32.29	160	7	28	hsa-miR-7851-3p	SEPT7P3	-25.42	164	19279	19300	SEPT7P3	1	pseudo
AKT3	9	protein-coding	hsa-miR-7851-3p	AKT3	-32.29	160	7	28	hsa-miR-7851-3p	SEPT7P3	-28.88	160	18093	18114	SEPT7P3	1	pseudo
AKT3	9	protein-coding	hsa-miR-7851-3p	AKT3	-32.29	160	7	28	hsa-miR-7851-3p	LOC100420851	-37.6	192	40752	40773	LOC100420851	1	pseudo
AKT3	9	protein-coding	hsa-miR-7851-3p	AKT3	-32.29	160	7	28	hsa-miR-7851-3p	LOC100420851	-34.02	184	23347	23368	LOC100420851	1	pseudo
AKT3	9	protein-coding	hsa-miR-7851-3p	AKT3	-32.29	160	7	28	hsa-miR-7851-3p	LOC100420851	-35.62	178	23379	23400	LOC100420851	1	pseudo
AKT3	9	protein-coding	hsa-miR-7851-3p	AKT3	-32.29	160	7	28	hsa-miR-7851-3p	LOC100420851	-29.06	172	62760	62781	LOC100420851	1	pseudo
AKT3	9	protein-coding	hsa-miR-7851-3p	AKT3	-32.29	160	7	28	hsa-miR-7851-3p	KCNQ1OT1	-35.47	170	45438	45458	KCNQ1OT1	9	ncRNA
AKT3	9	protein-coding	hsa-miR-7851-3p	AKT3	-32.29	160	7	28	hsa-miR-7851-3p	KCNQ1OT1	-27.52	161	18209	18230	KCNQ1OT1	9	ncRNA
AKT3	9	protein-coding	hsa-miR-7851-3p	AKT3	-32.29	160	7	28	hsa-miR-7851-3p	HERC2P5	-32.3	168	2154	2175	HERC2P5	1	pseudo
AKT3	9	protein-coding	hsa-miR-7851-3p	AKT3	-32.29	160	7	28	hsa-miR-7851-3p	HERC2P5	-30.12	168	3289	3310	HERC2P5	1	pseudo
AKT3	9	protein-coding	hsa-miR-7851-3p	AKT3	-32.29	160	7	28	hsa-miR-7851-3p	HERC2P5	-27.47	160	5829	5850	HERC2P5	1	pseudo
AKT3	9	protein-coding	hsa-miR-7851-3p	AKT3	-32.29	160	7	28	hsa-miR-7851-3p	HERC2P5	-31.35	160	21720	21741	HERC2P5	1	pseudo
AKT3	9	protein-coding	hsa-miR-7851-3p	AKT3	-32.29	160	7	28	hsa-miR-7851-3p	EP400NL	-37.91	192	209	230	EP400NL	1	pseudo
AKT3	9	protein-coding	hsa-miR-7851-3p	AKT3	-32.29	160	7	28	hsa-miR-7851-3p	LINC01021	-28.55	160	709	730	LINC01021	1	ncRNA
AKT3	9	protein-coding	hsa-miR-7851-3p	AKT3	-32.29	160	7	28	hsa-miR-7851-3p	GAS6-AS2	-27.47	160	175	196	GAS6-AS2	1	ncRNA
AKT3	9	protein-coding	hsa-miR-7851-3p	AKT3	-32.29	160	7	28	hsa-miR-7851-3p	LOC155060	-27.93	160	1060	1081	LOC155060	1	pseudo
AKT3	9	protein-coding	hsa-miR-7851-3p	AKT3	-32.29	160	7	28	hsa-miR-7851-3p	LOC101928139	-31.42	160	770	788	LOC101928139	1	ncRNA
AKT3	9	protein-coding	hsa-miR-7851-3p	AKT3	-32.29	160	7	28	hsa-miR-7851-3p	LOC440300	-32.08	164	6886	6907	LOC440300	1	pseudo
AKT3	9	protein-coding	hsa-miR-7851-3p	AKT3	-32.29	160	7	28	hsa-miR-7851-3p	LOC101928042	-27.17	168	5366	5387	LOC101928042	0	ncRNA
AKT3	9	protein-coding	hsa-miR-7851-3p	AKT3	-32.29	160	7	28	hsa-miR-7851-3p	MIRLET7BHG	-26.92	163	742	764	MIRLET7BHG	1	ncRNA
AKT3	9	protein-coding	hsa-miR-7851-3p	AKT3	-32.29	160	7	28	hsa-miR-7851-3p	SEPSECS-AS1	-37.25	176	136	157	SEPSECS-AS1	0	ncRNA
AKT3	9	protein-coding	hsa-miR-7851-3p	AKT3	-32.29	160	7	28	hsa-miR-7851-3p	UBE2Q2P6	-34.02	184	5222	5243	UBE2Q2P6	1	pseudo
AKT3	9	protein-coding	hsa-miR-7851-3p	AKT3	-32.29	160	7	28	hsa-miR-7851-3p	UBE2Q2P6	-26.13	167	1301	1322	UBE2Q2P6	1	pseudo
AKT3	9	protein-coding	hsa-miR-7851-3p	AKT3	-32.29	160	7	28	hsa-miR-7851-3p	LOC100287825	-37.9	192	5064	5085	LOC100287825	9	pseudo
AKT3	9	protein-coding	hsa-miR-7851-3p	AKT3	-32.29	160	7	28	hsa-miR-7851-3p	LOC100287825	-37.18	164	85	106	LOC100287825	9	pseudo

AKT3	9	protein-coding	hsa-miR-7851-3p	AKT3	-32.29	160	7	28	hsa-miR-7851-3p	FAM115B	-28.32	168	8635	8656	FAM115B	0	pseudo
AKT3	9	protein-coding	hsa-miR-7851-3p	AKT3	-32.29	160	7	28	hsa-miR-7851-3p	LOC100506023	-35.13	160	580	601	LOC100506023	0	ncRNA
AKT3	9	protein-coding	hsa-miR-7851-3p	AKT3	-32.29	160	7	28	hsa-miR-7851-3p	LINC00476	-31.19	168	3879	3900	LINC00476	0	ncRNA
AKT3	9	protein-coding	hsa-miR-7851-3p	AKT3	-32.29	160	7	28	hsa-miR-7851-3p	ASMTL-AS1	-27.47	160	1915	1936	ASMTL-AS1	9	ncRNA
AKT3	9	protein-coding	hsa-miR-7851-3p	AKT3	-32.29	160	7	28	hsa-miR-7851-3p	FAM86HP	-33.72	164	1371	1392	FAM86HP	1	pseudo
AKT3	9	protein-coding	hsa-miR-7851-3p	AKT3	-32.29	160	7	28	hsa-miR-7851-3p	CROCCP3	-32.95	184	363	384	CROCCP3	0	pseudo
AKT3	9	protein-coding	hsa-miR-7851-3p	AKT3	-32.29	160	7	28	hsa-miR-7851-3p	RBMS1P1	-27.47	160	1768	1789	RBMS1P1	0	pseudo
AKT3	9	protein-coding	hsa-miR-4745-3p	AKT3	-34.76	171	205	229	hsa-miR-4745-3p	LOC100126784	-27.98	161	1052	1074	LOC100126784	1	ncRNA
AKT3	9	protein-coding	hsa-miR-4745-3p	AKT3	-34.76	171	205	229	hsa-miR-4745-3p	SH3GL1P1	-25.24	163	716	738	SH3GL1P1	0	pseudo
AKT3	9	protein-coding	hsa-miR-4745-3p	AKT3	-34.76	171	205	229	hsa-miR-4745-3p	HERC2P2	-30.2	160	89	109	HERC2P2	9	pseudo
AKT3	9	protein-coding	hsa-miR-4745-3p	AKT3	-34.76	171	205	229	hsa-miR-4745-3p	LINC01001	-28.49	163	1931	1952	LINC01001	9	ncRNA
AKT3	9	protein-coding	hsa-miR-4745-3p	AKT3	-34.76	171	205	229	hsa-miR-4745-3p	LINC01001	-27.93	161	1993	2015	LINC01001	9	ncRNA
AKT3	9	protein-coding	hsa-miR-4745-3p	AKT3	-34.76	171	205	229	hsa-miR-4745-3p	LINC01000	-29.59	165	2204	2226	LINC01000	1	ncRNA
AKT3	9	protein-coding	hsa-miR-4745-3p	AKT3	-34.76	171	205	229	hsa-miR-4745-3p	LINC01000	-28.49	163	2142	2163	LINC01000	1	ncRNA
AKT3	9	protein-coding	hsa-miR-4745-3p	AKT3	-34.76	171	205	229	hsa-miR-4745-3p	LINC01000	-31.49	163	3262	3285	LINC01000	1	ncRNA
AKT3	9	protein-coding	hsa-miR-4745-3p	AKT3	-34.76	171	205	229	hsa-miR-4745-3p	LOC100129321	-30.14	166	465	486	LOC100129321	9	pseudo
AKT3	9	protein-coding	hsa-miR-4745-3p	AKT3	-34.76	171	205	229	hsa-miR-4745-3p	HOXB-AS3	-36.3	160	49	72	HOXB-AS3	1	ncRNA
AKT3	9	protein-coding	hsa-miR-4745-3p	AKT3	-34.76	171	205	229	hsa-miR-4745-3p	LOC202181	-34.63	166	198	219	LOC202181	9	pseudo
AKT3	9	protein-coding	hsa-miR-4745-3p	AKT3	-34.76	171	205	229	hsa-miR-4745-3p	LOC100506023	-36.4	168	26	50	LOC100506023	0	ncRNA
AKT3	9	protein-coding	hsa-miR-4745-3p	AKT3	-34.76	171	205	229	hsa-miR-4745-3p	SEC14L1P1	-34.07	171	1982	2005	SEC14L1P1	0	pseudo
AKT3	9	protein-coding	hsa-miR-6848-5p	AKT3	-27.95	160	33	55	hsa-miR-6848-5p	KIAA1656	-39.33	163	2298	2322	KIAA1656	1	ncRNA
AKT3	9	protein-coding	hsa-miR-6848-5p	AKT3	-27.95	160	33	55	hsa-miR-6848-5p	ERLEC1P1	-39.88	169	5325	5347	ERLEC1P1	1	pseudo
AKT3	9	protein-coding	hsa-miR-6848-5p	AKT3	-27.95	160	33	55	hsa-miR-6848-5p	LOC101060398	-31.66	160	621	643	LOC101060398	9	ncRNA
AKT3	9	protein-coding	hsa-miR-6848-5p	AKT3	-27.95	160	33	55	hsa-miR-6848-5p	LINC00886	-29.48	160	1713	1735	LINC00886	1	ncRNA
AKT3	9	protein-coding	hsa-miR-6848-5p	AKT3	-27.95	160	33	55	hsa-miR-6848-5p	LOC100420851	-37.01	161	52261	52283	LOC100420851	1	pseudo
AKT3	9	protein-coding	hsa-miR-6848-5p	AKT3	-27.95	160	33	55	hsa-miR-6848-5p	LOC100420851	-36.84	160	42895	42917	LOC100420851	1	pseudo
AKT3	9	protein-coding	hsa-miR-6848-5p	AKT3	-27.95	160	33	55	hsa-miR-6848-5p	KCNQ1OT1	-31.34	161	25909	25932	KCNQ1OT1	9	ncRNA
AKT3	9	protein-coding	hsa-miR-6848-5p	AKT3	-27.95	160	33	55	hsa-miR-6848-5p	CSPG4P12	-29.44	163	3518	3542	CSPG4P12	1	pseudo
AKT3	9	protein-coding	hsa-miR-6848-5p	AKT3	-27.95	160	33	55	hsa-miR-6848-5p	CSPG4P12	-35.35	160	105	129	CSPG4P12	1	pseudo
AKT3	9	protein-coding	hsa-miR-6848-5p	AKT3	-27.95	160	33	55	hsa-miR-6848-5p	CSPG4P10	-29.44	163	3493	3517	CSPG4P10	1	pseudo
AKT3	9	protein-coding	hsa-miR-6848-5p	AKT3	-27.95	160	33	55	hsa-miR-6848-5p	CSPG4P10	-35.35	160	79	103	CSPG4P10	1	pseudo
AKT3	9	protein-coding	hsa-miR-6848-5p	AKT3	-27.95	160	33	55	hsa-miR-6848-5p	CSPG4P11	-29.44	163	3580	3604	CSPG4P11	1	pseudo
AKT3	9	protein-coding	hsa-miR-6848-5p	AKT3	-27.95	160	33	55	hsa-miR-6848-5p	CSPG4P11	-35.35	160	148	172	CSPG4P11	1	pseudo
AKT3	9	protein-coding	hsa-miR-6848-5p	AKT3	-27.95	160	33	55	hsa-miR-6848-5p	KCNJ2-AS1	-32.74	166	371	393	KCNJ2-AS1	1	ncRNA
AKT3	9	protein-coding	hsa-miR-6848-5p	AKT3	-27.95	160	33	55	hsa-miR-6848-5p	LINC01000	-28.45	160	8462	8484	LINC01000	1	ncRNA

AKT3	9	protein-coding	hsa-miR-6848-5p	AKT3	-27.95	160	33	55	hsa-miR-6848-5p	LOC101929484	-30.2	160	262	285	LOC101929484	1	ncRNA
AKT3	9	protein-coding	hsa-miR-6848-5p	AKT3	-27.95	160	33	55	hsa-miR-6848-5p	MIRLET7BHG	-33.93	166	3354	3376	MIRLET7BHG	1	ncRNA
AKT3	9	protein-coding	hsa-miR-6848-5p	AKT3	-27.95	160	33	55	hsa-miR-6848-5p	LOC102724191	-29.44	163	1837	1861	LOC102724191	1	pseudo
AKT3	9	protein-coding	hsa-miR-6848-5p	AKT3	-27.95	160	33	55	hsa-miR-6848-5p	LOC102724191	-35.35	160	73	97	LOC102724191	1	pseudo
AKT3	9	protein-coding	hsa-miR-6848-5p	AKT3	-27.95	160	33	55	hsa-miR-6848-5p	SND1-IT1	-35.97	161	1178	1200	SND1-IT1	9	ncRNA
AKT3	9	protein-coding	hsa-miR-6848-5p	AKT3	-27.95	160	33	55	hsa-miR-6848-5p	LOC100287825	-28.12	161	5793	5815	LOC100287825	9	pseudo
AKT3	9	protein-coding	hsa-miR-6848-5p	AKT3	-27.95	160	33	55	hsa-miR-6848-5p	CYP1B1-AS1	-29.23	162	1191	1211	CYP1B1-AS1	9	ncRNA
AKT3	9	protein-coding	hsa-miR-6848-5p	AKT3	-27.95	160	33	55	hsa-miR-6848-5p	ERVK13-1	-36.31	161	1772	1794	ERVK13-1	1	ncRNA
AKT3	9	protein-coding	hsa-miR-6848-5p	AKT3	-27.95	160	33	55	hsa-miR-6848-5p	RBMS1P1	-43.94	161	30	52	RBMS1P1	0	pseudo
AKT3	9	protein-coding	hsa-miR-1273e	AKT3	-28.91	160	14	35	hsa-miR-1273e	LOC100506990	-34.07	168	1693	1714	LOC100506990	9	ncRNA
AKT3	9	protein-coding	hsa-miR-1273e	AKT3	-28.91	160	14	35	hsa-miR-1273e	RHOQP2	-34.38	184	5013	5034	RHOQP2	9	pseudo
AKT3	9	protein-coding	hsa-miR-1273e	AKT3	-28.91	160	14	35	hsa-miR-1273e	RHOQP2	-33.6	164	14604	14625	RHOQP2	9	pseudo
AKT3	9	protein-coding	hsa-miR-1273e	AKT3	-28.91	160	14	35	hsa-miR-1273e	ERLEC1P1	-31.3	184	2928	2949	ERLEC1P1	1	pseudo
AKT3	9	protein-coding	hsa-miR-1273e	AKT3	-28.91	160	14	35	hsa-miR-1273e	ERLEC1P1	-31.02	180	15353	15374	ERLEC1P1	1	pseudo
AKT3	9	protein-coding	hsa-miR-1273e	AKT3	-28.91	160	14	35	hsa-miR-1273e	ERLEC1P1	-30.52	176	10175	10196	ERLEC1P1	1	pseudo
AKT3	9	protein-coding	hsa-miR-1273e	AKT3	-28.91	160	14	35	hsa-miR-1273e	ERLEC1P1	-29.11	173	22412	22432	ERLEC1P1	1	pseudo
AKT3	9	protein-coding	hsa-miR-1273e	AKT3	-28.91	160	14	35	hsa-miR-1273e	ERLEC1P1	-25.67	168	18393	18414	ERLEC1P1	1	pseudo
AKT3	9	protein-coding	hsa-miR-1273e	AKT3	-28.91	160	14	35	hsa-miR-1273e	ERLEC1P1	-28.32	163	22276	22297	ERLEC1P1	1	pseudo
AKT3	9	protein-coding	hsa-miR-1273e	AKT3	-28.91	160	14	35	hsa-miR-1273e	UBE2Q2P12	-39.54	192	2950	2971	UBE2Q2P12	1	pseudo
AKT3	9	protein-coding	hsa-miR-1273e	AKT3	-28.91	160	14	35	hsa-miR-1273e	UBE2Q2P12	-25.7	168	1342	1363	UBE2Q2P12	1	pseudo
AKT3	9	protein-coding	hsa-miR-1273e	AKT3	-28.91	160	14	35	hsa-miR-1273e	UBE2Q2P11	-39.54	192	2947	2968	UBE2Q2P11	1	pseudo
AKT3	9	protein-coding	hsa-miR-1273e	AKT3	-28.91	160	14	35	hsa-miR-1273e	UBE2Q2P11	-25.7	168	1328	1349	UBE2Q2P11	1	pseudo
AKT3	9	protein-coding	hsa-miR-1273e	AKT3	-28.91	160	14	35	hsa-miR-1273e	LOC100130872	-27.26	176	2782	2803	LOC100130872	1	ncRNA
AKT3	9	protein-coding	hsa-miR-1273e	AKT3	-28.91	160	14	35	hsa-miR-1273e	SEPT7P3	-32.61	184	17208	17229	SEPT7P3	1	pseudo
AKT3	9	protein-coding	hsa-miR-1273e	AKT3	-28.91	160	14	35	hsa-miR-1273e	SEPT7P3	-26.82	176	5318	5339	SEPT7P3	1	pseudo
AKT3	9	protein-coding	hsa-miR-1273e	AKT3	-28.91	160	14	35	hsa-miR-1273e	SEPT7P3	-37.74	172	7508	7529	SEPT7P3	1	pseudo
AKT3	9	protein-coding	hsa-miR-1273e	AKT3	-28.91	160	14	35	hsa-miR-1273e	LOC100420851	-25.54	173	48299	48319	LOC100420851	1	pseudo
AKT3	9	protein-coding	hsa-miR-1273e	AKT3	-28.91	160	14	35	hsa-miR-1273e	LOC100420851	-25.36	168	23354	23375	LOC100420851	1	pseudo
AKT3	9	protein-coding	hsa-miR-1273e	AKT3	-28.91	160	14	35	hsa-miR-1273e	LOC100420851	-30.46	160	62767	62788	LOC100420851	1	pseudo
AKT3	9	protein-coding	hsa-miR-1273e	AKT3	-28.91	160	14	35	hsa-miR-1273e	KCNQ1OT1	-34.72	184	51860	51881	KCNQ1OT1	9	ncRNA
AKT3	9	protein-coding	hsa-miR-1273e	AKT3	-28.91	160	14	35	hsa-miR-1273e	HERC2P5	-32.61	184	2616	2637	HERC2P5	1	pseudo
AKT3	9	protein-coding	hsa-miR-1273e	AKT3	-28.91	160	14	35	hsa-miR-1273e	HERC2P5	-33.8	180	2161	2182	HERC2P5	1	pseudo
AKT3	9	protein-coding	hsa-miR-1273e	AKT3	-28.91	160	14	35	hsa-miR-1273e	HERC2P5	-32.24	168	21727	21748	HERC2P5	1	pseudo
AKT3	9	protein-coding	hsa-miR-1273e	AKT3	-28.91	160	14	35	hsa-miR-1273e	LINC01000	-44.26	200	8254	8275	LINC01000	1	ncRNA
AKT3	9	protein-coding	hsa-miR-1273e	AKT3	-28.91	160	14	35	hsa-miR-1273e	LOC440300	-28.03	173	6199	6219	LOC440300	1	pseudo

AKT3	9	protein-coding	hsa-miR-1273e	AKT3	-28.91	160	14	35	hsa-miR-1273e	LOC440300	-25.18	168	2012	2033	LOC440300	1	pseudo
AKT3	9	protein-coding	hsa-miR-1273e	AKT3	-28.91	160	14	35	hsa-miR-1273e	LOC101929130	-39.48	176	1614	1635	LOC101929130	1	ncRNA
AKT3	9	protein-coding	hsa-miR-1273e	AKT3	-28.91	160	14	35	hsa-miR-1273e	UBE2Q2P6	-39.54	192	2929	2950	UBE2Q2P6	1	pseudo
AKT3	9	protein-coding	hsa-miR-1273e	AKT3	-28.91	160	14	35	hsa-miR-1273e	UBE2Q2P6	-25.7	168	1308	1329	UBE2Q2P6	1	pseudo
AKT3	9	protein-coding	hsa-miR-1273e	AKT3	-28.91	160	14	35	hsa-miR-1273e	LINC00346	-32.41	180	1712	1733	LINC00346	1	ncRNA
AKT3	9	protein-coding	hsa-miR-1273e	AKT3	-28.91	160	14	35	hsa-miR-1273e	HTR7P1	-31.91	168	3552	3573	HTR7P1	0	pseudo
AKT3	9	protein-coding	hsa-miR-1273e	AKT3	-28.91	160	14	35	hsa-miR-1273e	LOC100506023	-33.24	180	587	608	LOC100506023	0	ncRNA
AKT3	9	protein-coding	hsa-miR-1273e	AKT3	-28.91	160	14	35	hsa-miR-1273e	ASMTL-AS1	-33.6	164	1922	1943	ASMTL-AS1	9	ncRNA
AKT3	9	protein-coding	hsa-miR-1273e	AKT3	-28.91	160	14	35	hsa-miR-1273e	ERVK13-1	-34.07	168	413	434	ERVK13-1	1	ncRNA
AKT3	9	protein-coding	hsa-miR-3159	AKT3	-26.66	160	584	605	hsa-miR-3159	ERLEC1P1	-29.97	160	8954	8975	ERLEC1P1	1	pseudo
AKT3	9	protein-coding	hsa-miR-3159	AKT3	-26.66	160	584	605	hsa-miR-3159	ERLEC1P1	-25.67	160	26913	26934	ERLEC1P1	1	pseudo
AKT3	9	protein-coding	hsa-miR-3159	AKT3	-26.66	160	584	605	hsa-miR-3159	ERLEC1P1	-26.23	160	34944	34965	ERLEC1P1	1	pseudo
AKT3	9	protein-coding	hsa-miR-3159	AKT3	-26.66	160	584	605	hsa-miR-3159	NPHP3-ACAD11	-29.01	160	7272	7293	NPHP3-ACAD11	9	ncRNA
AKT3	9	protein-coding	hsa-miR-3159	AKT3	-26.66	160	584	605	hsa-miR-3159	LOC100420851	-26.23	160	30419	30440	LOC100420851	1	pseudo
AKT3	9	protein-coding	hsa-miR-3159	AKT3	-26.66	160	584	605	hsa-miR-3159	LOC100420851	-26.23	160	54890	54911	LOC100420851	1	pseudo
AKT3	9	protein-coding	hsa-miR-3159	AKT3	-26.66	160	584	605	hsa-miR-3159	KCNQ1OT1	-26.23	160	88499	88520	KCNQ1OT1	9	ncRNA
AKT3	9	protein-coding	hsa-miR-3159	AKT3	-26.66	160	584	605	hsa-miR-3159	KCNQ1OT1	-26.23	160	91222	91243	KCNQ1OT1	9	ncRNA
AKT3	9	protein-coding	hsa-miR-3159	AKT3	-26.66	160	584	605	hsa-miR-3159	ASAP1-IT2	-26.23	160	1923	1944	ASAP1-IT2	9	ncRNA
AKT3	9	protein-coding	hsa-miR-3159	AKT3	-26.66	160	584	605	hsa-miR-3159	PRKY	-27.14	180	3536	3557	PRKY	1	pseudo
AKT3	9	protein-coding	hsa-miR-3159	AKT3	-26.66	160	584	605	hsa-miR-3159	ZNF702P	-26.23	160	2457	2478	ZNF702P	1	pseudo
AKT3	9	protein-coding	hsa-miR-3159	AKT3	-26.66	160	584	605	hsa-miR-3159	LOC653406	-26.23	160	3875	3896	LOC653406	1	pseudo
AKT3	9	protein-coding	hsa-miR-3159	AKT3	-26.66	160	584	605	hsa-miR-3159	LOC440300	-26.23	160	4324	4345	LOC440300	1	pseudo
AKT3	9	protein-coding	hsa-miR-3159	AKT3	-26.66	160	584	605	hsa-miR-3159	ZNF37BP	-26.06	160	5913	5934	ZNF37BP	9	pseudo
AKT3	9	protein-coding	hsa-miR-3159	AKT3	-26.66	160	584	605	hsa-miR-3159	KLF3P1	-28.45	160	1488	1509	KLF3P1	0	pseudo
AKT3	9	protein-coding	hsa-miR-3159	AKT3	-26.66	160	584	605	hsa-miR-3159	LOC728519	-26.23	160	4538	4559	LOC728519	1	pseudo

Supplementary Table 3.

Primer sequences for polymerase chain reaction amplification.

mRNA	Primer sequence
α-SMA	Sense 5-CGGGACATCAAGGAGAAACT-3
	Antisence 5-CCCATCAGGCAACTCGTAA-3
vimentin	Sense 5-CCAAACTTTTCCTCCCTGAACC-3
	Antisence 5-GTGATGCTGAGAAGTTTCGTTGA-3
K6	Sense 5-CAAGTCAACATCTCTGTGGTGC-3
	Antisence 5-TGGGACCGAGAGCTAGCAG-3
K19	Sense 5- GAAGGATGCTGAAGCCTGGT-3
	Antisence 5- CTGGGCTTCAATACCGCTGA-3
KIAA1656	Sense 5-AGCGAGGAGTAAGCATCAGAGG-3
	Antisence 5-GCAAGGAGCCAGGAGTTCAGTT-3
LINC00672	Sense 5-GCGAAGAAGGCAGTCAGGAGGA-3
	Antisence 5-ACCAACCACAGCCAACCAATCAC-3
LOC102724927	Sense 5-GCAGCCACCAGAAGGAATGAGA-3
	Antisence 5-CCCGCATCCCGCATACATAGAT-3
ASAP1-IT2	Sense 5-CCAGCACGGTCAGTCCTGTCTT-3
	Antisence 5-TGATCCACCTGCCTCGGTCTCT-3
MIRLET7BHG	Sense 5-GCTGCGAGTATTGGCGTTGC-3
	Antisence 5-GCTGTTCCTCTCACTTCCTGCT-3
LINC01000	Sense 5-TCTGCCTCACAGCGGACTCT-3
	Antisence 5-GGAGACGCAACCAGGAAGAAGA-3
miR-619-5P	Sense 5-GCUGGGAUUACAGGCAUGAGCC-3
	Antisence 5-TGCTGTCAACGATACGCTACG-3
ACTB	Sense 5-CATGTACGTTGCTATCCAGGC-3
	Antisence 5-CTCCTTAATGTCACGCACGAT-3
FGF5	Sense 5-GCTGCCACTGATAGGAACCC-3
	Antisence 5-CCCCTGAGACACAGCAAATA-3
FGF7	Sense 5-ACAAAAGTCAAATAGCAAACA-3
	Antisence 5-ATGTCAGTATCCATTTGTGC-3
AKT3	Sense 5-CTTATCCCCTCAACAACTTTTC-3

	Antisence 5-GCTTCTGTCCATTCTTCCCT-3
TLR4	Sense 5-CGATTCCATTGCTTCTTGCT-3
	Antisence 5-GAGGTGGCTTAGGCTCTGATA-3
IL-6	Sense 5-TCAATATTAGAGTCTCAACCCCCA-3
	Antisence 5- GAAGGCGCTTGTGGAGAAGG-3
